# Monitoring calcium handling by the plant endoplasmic reticulum with a low‐Ca^2+^‐affinity targeted aequorin reporter

**DOI:** 10.1111/tpj.15610

**Published:** 2021-12-11

**Authors:** Enrico Cortese, Roberto Moscatiello, Francesca Pettiti, Luca Carraretto, Barbara Baldan, Lorenzo Frigerio, Ute C. Vothknecht, Ildiko Szabo, Diego De Stefani, Marisa Brini, Lorella Navazio

**Affiliations:** ^1^ Department of Biology University of Padova Padova 35131 Italy; ^2^ Botanical Garden University of Padova Padova 35123 Italy; ^3^ School of Life Sciences University of Warwick Coventry CV4 7AL UK; ^4^ Plant Cell Biology Institute of Cellular and Molecular Botany University of Bonn Bonn D‐53115 Germany; ^5^ Department of Biomedical Sciences University of Padova Padova 35131 Italy

**Keywords:** calcium homeostasis, endoplasmic reticulum, chloroplasts, aequorin, *Arabidopsis thaliana*, signal transduction, environmental stresses

## Abstract

Precise measurements of dynamic changes in free Ca^2+^ concentration in the lumen of the plant endoplasmic reticulum (ER) have been lacking so far, despite increasing evidence for the contribution of this intracellular compartment to Ca^2+^ homeostasis and signalling in the plant cell. In the present study, we targeted an aequorin chimera with reduced Ca^2+^ affinity to the ER membrane and facing the ER lumen. To this aim, the cDNA for a low‐Ca^2+^‐affinity aequorin variant (AEQmut) was fused to the nucleotide sequence encoding a non‐cleavable N‐terminal ER signal peptide (fl2). The correct targeting of fl2‐AEQmut was confirmed by immunocytochemical analyses in transgenic *Arabidopsis thaliana* (Arabidopsis) seedlings. An experimental protocol well‐established in animal cells – consisting of ER Ca^2+^ depletion during photoprotein reconstitution followed by ER Ca^2+^ refilling – was applied to carry out ER Ca^2+^ measurements *in planta*. Rapid and transient increases of the ER luminal Ca^2+^ concentration ([Ca^2+^]_ER_) were recorded in response to different environmental stresses, displaying stimulus‐specific Ca^2+^ signatures. The comparative analysis of ER and chloroplast Ca^2+^ dynamics indicates a complex interplay of these organelles in shaping cytosolic Ca^2+^ signals during signal transduction events. Our data highlight significant differences in basal [Ca^2+^]_ER_ and Ca^2+^ handling by plant ER compared to the animal counterpart. The set‐up of an ER‐targeted aequorin chimera extends and complements the currently available toolkit of organelle‐targeted Ca^2+^ indicators by adding a reporter that improves our quantitative understanding of Ca^2+^ homeostasis in the plant endomembrane system.

## Significance Statement

A better understanding of Ca^2+^ homeostasis in the plant endoplasmic reticulum (ER) requires accurate measurements of the free Ca^2+^ concentration in the lumen of this compartment. We designed a specifically ER‐targeted aequorin chimera that allowed quantitative monitoring of dynamic changes in ER Ca^2+^ levels in Arabidopsis seedlings in response to environmental cues and we analysed the interplay of ER and chloroplasts in shaping cytosolic Ca^2+^ signals.

## INTRODUCTION

Calcium is a fundamental intracellular messenger that plays a key role in the transduction of a wide range of stimuli in all living organisms (Berridge et al., 2000; Dodd et al., 2010; Domínguez et al., 2015). The expanding field of organellar Ca^2+^ signalling has led to an ever‐increasing knowledge of how different intracellular compartments of eukaryotic cells contribute to orchestrating complex and specific Ca^2+^‐mediated responses (Brini et al., 2013; Costa et al., 2018; Pirayesh et al., 2021; Resentini et al., 2021a; Stael et al., 2012). Implementation of Ca^2+^ reporter‐based technologies (Alonso et al., 2017; Pérez Koldenkova and Nagai, 2013) into cellular and molecular studies has helped to provide a more detailed picture of the complexity of intracellular Ca^2+^ signalling networks, paving the way to investigate the fine‐tuned integration of internal mobilizable Ca^2+^ stores in achieving an efficient Ca^2+^ homeostasis and signal transduction (Brini et al., 2013; Costa et al., 2018). Nevertheless, information on the precise role of different intracellular compartments in Ca^2+^ handling in the plant cell is still incomplete, especially regarding compartments such as the endoplasmic reticulum (ER), for which only putative estimates of basal [Ca^2+^] and its changes are available (Costa et al., 2018; Stael et al., 2012).

In mammalian cells, reports of free [Ca^2+^] in the ER lumen ([Ca^2+^]_ER_) range from 50 to 500 μm (Coe and Michalak, 2009) and research studies conducted in the last 25 years have highlighted the role played by this compartment as a major intracellular Ca^2+^ store (Meldolesi and Pozzan, 1998; Montero et al., 1995; Raffaello et al., 2016; Wang et al., 2019).

Comparatively less information is available on the Ca^2+^ storage properties of the plant ER, mainly relating to the presence of Ca^2+^ buffering proteins in the ER lumen – namely calreticulin, for which the high‐capacity and low‐affinity Ca^2+^‐binding properties indicate a sub‐millimolar [Ca^2+^]_ER_ (Joshi et al., 2019; Mariani et al., 2003) – and from circumstantial estimates carried out with cameleon‐based Ca^2+^ indicators (Bonza et al., 2013; Iwano et al., 2009). Concerning Ca^2+^ transporters localized at the ER membrane, two distinct types of Ca^2+^‐ATPases have been identified: P‐type IIA ER‐type Ca^2+^‐ATPases (ECAs) and P‐type IIB autoinhibited Ca^2+^‐ATPases (ACAs) (Bonza and De Michelis, 2011; García Bossi et al., 2020). In particular, ECA1 has proven to be fundamental for proper ER Ca^2+^ homeostasis because treatment with its specific blocker cyclopiazonic acid (CPA) led to a reduced [Ca^2+^] in the ER lumen, with a parallel increase in the cytosolic Ca^2+^ pool (Bonza et al., 2013; Zuppini et al., 2004). More recently, the ER‐located Ca^2+^/cation exchanger CCX2 has been reported to be involved in Ca^2+^‐mediated signal transduction triggered by osmotic stress (Corso et al., 2018). Conversely, no Ca^2+^‐permeable channels have been identified yet in the plant ER membrane, despite biochemical evidence for the occurrence of voltage‐gated (Klüsener et al., 1995) and ligand‐gated (Navazio et al., 2000, 2001) Ca^2+^ mobilization pathways involved in Ca^2+^ fluxes between the ER and cytosol. Because of its continuity with the nuclear outer membrane, the ER may play a key role in the modulation of nucleus‐associated Ca^2+^ oscillations during nitrogen‐fixing and mycorrhizal symbioses (Capoen et al., 2011; Charpentier et al., 2016), as well as in Ca^2+^ signalling events that extend beyond plant‐microbe symbioses, such as root development (Leitão et al., 2019). The ER is also known to make multiple contacts with other intracellular compartments, through which Ca^2+^ fluxes may occur. Moreover, spatially confined anchor sites between the plant cortical ER and the plasma membrane (PM) have been detected (Bayer et al., 2017; Wang et al., 2014). In animal cells ER–PM contact sites are known to be crucial for lipid transfer, as well as for modulating cytosolic Ca^2+^ signals through Ca^2+^ release from the ER (Saheki and De Camilli, 2017) and subsequent ER reload (Chung et al., 2017). A distinctive feature of plant cells is the occurrence of interactions between the ER and stromules, stroma‐filled protrusions stemming from both green and non‐green plastids. These organelle projections were observed to extend and retract from the plastid body in an ER‐aided manner (Schattat et al., 2011) and were demonstrated to be the site of lipid exchange (Block and Jouhet, 2015; Liu and Li, 2019). The possible occurrence of ion fluxes at these contact sites (e.g. a potential ER‐plastid crosstalk in terms of Ca^2+^ handling) adds a further level of complexity to the already intricate plant Ca^2+^ signalling scenario (Mehrshahi et al., 2013).

In the present study, we developed a novel Ca^2+^ reporter for the plant ER that is useful for quantitative analyses of Ca^2+^ signatures in this compartment. We fused a mutated version of the Ca^2+^‐sensitive photoprotein aequorin, characterized by a reduced Ca^2+^ affinity, to a non‐cleavable ER signal peptide. This targeting strategy allowed us to quantitatively monitor changes in [Ca^2+^]_ER_ in transgenic *Arabidopsis thaliana* (Arabidopsis) seedlings during stress‐related Ca^2+^ signal transduction events. The relative contribution of ER and chloroplasts in shaping cytosolic Ca^2+^ signals was also investigated.

The set‐up of a novel tool to quantitatively monitor [Ca^2+^]_ER_ in plant cells paves the way for future studies aimed at unravelling the integration of the plant ER in Ca^2+^ transport and signalling circuits.

## RESULTS

### Targeting an aequorin probe to the plant endoplasmic reticulum

The nucleotide sequence encoding a non‐cleavable N‐terminal ER signal peptide, responsible for the retention of an α‐zein storage protein in the ER of the maize mutant *floury2* (fl2) (Coleman et al., 1995; Gillikin et al., 1997), was fused to the cDNA for a mutated aequorin variant (AEQmut), characterized by a point substitution (D119A) leading to a reduced Ca^2+^ affinity (Montero et al., 1995). AEQmut had previously been used as a suitable Ca^2+^ probe to quantitatively measure ER Ca^2+^ levels ([Ca^2+^]_ER_) in mammalian cells (Brini, 2008; Montero et al., 1995; Ottolini et al., 2014).

**Figure 1:**
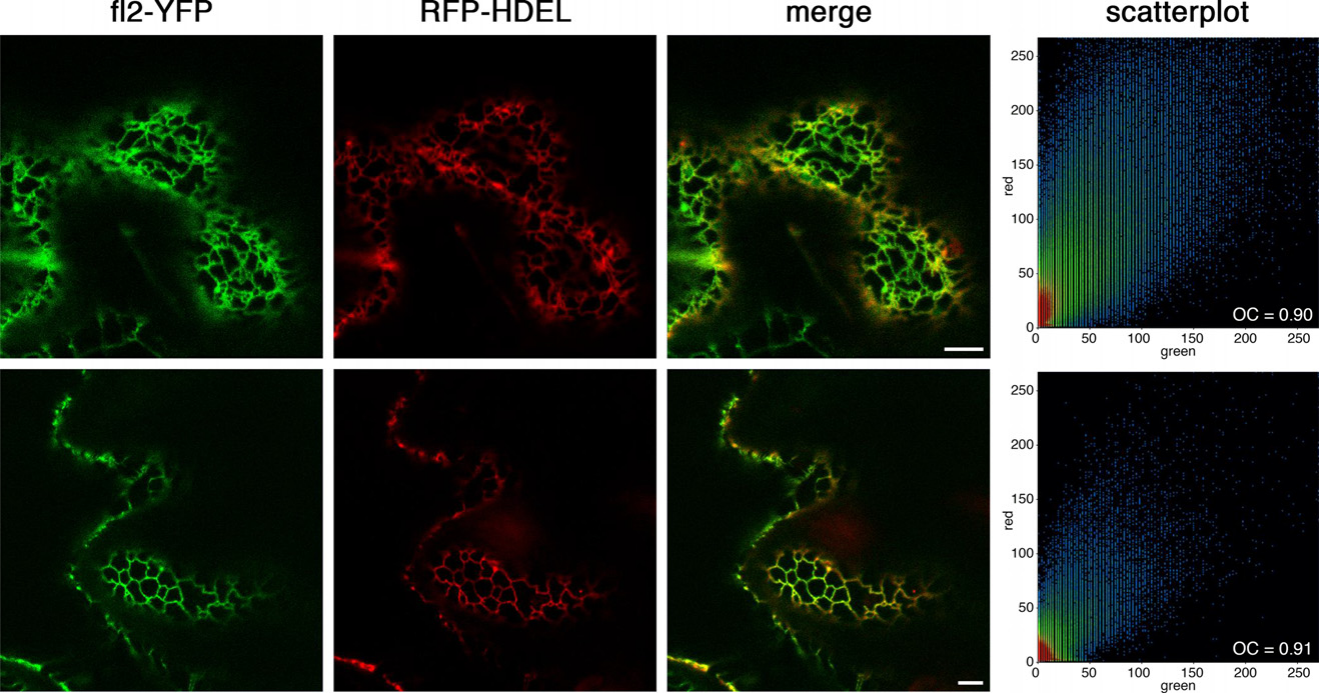
Subcellular localization of fl2‐YFP in *Nicotiana benthamiana*. Confocal microscopy analysis of *N. benthamiana* agroinfiltrated epidermal cells co‐transformed with fl2‐YFP and the ER marker RFP‐HDEL. Fluorescence microscopy images with filters for YFP, RFP and an overlay of the two channels are shown. Scale bar = 5 μm. Scatterplots of colocalization of the signals from the two constructs are also shown. OC, overlay coefficient.

**Figure 2:**
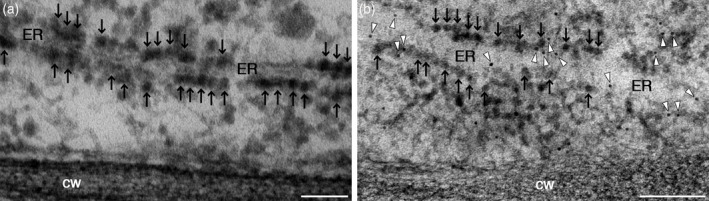
Subcellular localization of the fl2‐AEQmut Ca^2+^ probe in transgenic Arabidopsis seedlings. Immunogold labelling was carried out in roots of 2‐week‐old Arabidopsis seedlings stably transformed with fl2‐AEQmut using an anti‐aequorin antibody (dilution 1:500) followed by a secondary antibody conjugated with 10‐nm diameter gold particles (b). White arrowheads indicate gold particles, decorating ER profiles. Black arrows indicate ribosomes. As a negative control, samples were incubated with secondary antibody only (a). cw, cell wall. ER, endoplasmic reticulum. Scale bars = 100 nm.

The construct encoding the aequorin chimera fl2‐AEQmut was cloned in an expression cassette under the control of the 35S CaMV promoter in the pGreen 0029 plasmid (Figure S1a) and used for *Agrobacterium*‐mediated transformation of *A. thaliana* (Arabidopsis) via the floral dip method (Clough and Bent, 1998). After selection of the primary F_1_ transformants on kanamycin (50 μg ml^−1^), expression of the fl2‐AEQmut probe in transgenic Arabidopsis seedlings was checked by a reverse transcriptase‐polymerase chain reaction (RT‐PCR) and immunoblot analyses. Eleven out of 12 independent F_2_ lines were found to be positive at the level of aequorin gene expression (Figure S1b). The two lines (#6 and #10) showing the highest protein level (Figure S1c) were propagated (F_3_ generation) and used in the following analyses. No overall growth defects were observed in the ER‐targeted aequorin sensor lines (Figure S2a). Moreover, pulse amplitude modulated (PAM) imaging (Figure S2b) and transmission electron microscopy (TEM) analyses (Figure S2c) demonstrated the absence of any significant differences in the photosynthetic efficiency and cellular ultrastructural organization, respectively, of the transgenic lines compared to wild‐type plants. To test the efficiency of the selected ER‐targeting sequence, a construct encoding the fluorescent probe fl2‐YFP was generated and used for transient transformation of *Nicotiana benthamiana* leaves, as well as Arabidopsis leaves and protoplasts. In agroinfiltrated *N. benthamiana* leaves, the YFP fluorescence signal perfectly overlapped with the ER marker RFP‐HDEL (Figure 1). In transiently transformed Arabidopsis epidermal cells and protoplasts, the YFP signal was equally consistent with the distribution of ER membranes (Figure S3a). Confocal microscopy observations were also performed in stably transformed Arabidopsis seedlings, where the ER marker ER‐Tracker Red further confirmed the ER targeting of fl2‐fused YFP in the roots (Figure S3b). Likewise, a dynamic network of fluorescent tubules was clearly evident in the leaves, showing the typical, rapid remodelling of the ER membranes (Movie S1). In the case of the fl2‐AEQmut probe, the ER localization was ascertained by immunofluorescence (Figure S4) and immunogold labelling experiments (Figure 2) on Arabidopsis transgenic cell cultures and seedlings, respectively. TEM analyses showed the presence of electron‐dense gold particles in proximity of rough ER membranes (Figure 2).

### Set up of an efficient aequorin reconstitution protocol to enable *in vivo* Ca^2+^ measurements in the plant ER

Correct functioning of the plant ER‐targeted aequorin probe was first verified by *in vitro* reconstitution assays. Light emitted by total proteins contained in the lysates from Arabidopsis lines transformed with the fl2‐AEQmut construct was monitored after reconstitution of the apoprotein with coelenterazine. The luminescence signal detected in protein extracts from two independent transgenic lines confirmed the functionality of the reporter (Figure S5).

In the next step, intact fl2‐AEQmut Arabidopsis seedlings were challenged with a discharge solution [30% (v/v) ethanol, 1 m CaCl_2_] to evaluate the total emitted luminescence. The standard reconstitution protocol (overnight reconstitution with 5 μm coelenterazine), commonly employed in aequorin‐based Ca^2+^ assays (Sello et al., 2018; Teardo et al., 2019), was found to be unsuitable, because the recorded luminescence levels were too low for adequate Ca^2+^ measurements (Figure S6). To allow proper reconstitution of the fl2‐AEQmut probe, a preliminary step consisting in ER Ca^2+^ depletion was therefore added, in analogy with the experimental procedure commonly adopted for Ca^2+^ measurements in the animal ER (Brini, 2008; Montero et al., 1995; Ottolini et al., 2014). Different reconstitution procedures were tested, by applying the ionophore A23187 or the ER‐type Ca^2+^‐ATPase inhibitor CPA, either alone or together, in Ca^2+^‐free medium (600 µm EGTA) for 10 min prior to an incubation with 5 µm coelenterazine for an additional 2 h. A synthetic derivative of coelenterazine (coelenterazine *n*) that reduces the aequorin affinity for Ca^2+^, thereby lowering its rate of consumption in high [Ca^2+^] compartments (Ottolini et al., 2014), was also tested, as an alternative to wild‐type coelenterazine. However, coelenterazine *n* was found to drastically reduce the luminescence emitted by aequorin in all assays (Figure S6). Use of CPA, together with standard coelenterazine, provided the highest level of emitted luminescence (Figure S6) and was therefore chosen as the appropriate aequorin reconstitution protocol for the subsequent ER Ca^2+^ assays. Evans blue assays carried out in suspension‐cultured cells derived from the transgenic lines demonstrated that the adopted procedure did not affect cell viability (Figure S7).

**Figure 3:**
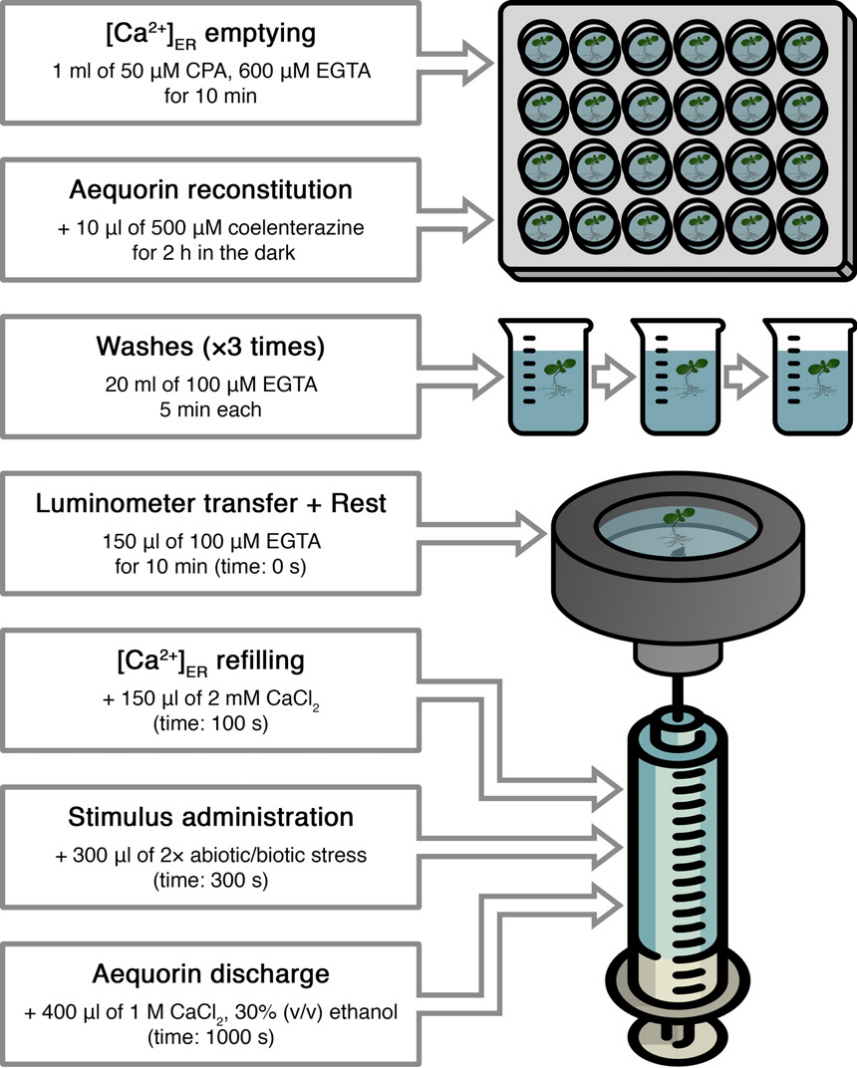
Schematic representation of the protocol used for Ca^2+^ measurements with the plant ER‐targeted aequorin probe.

In complying with the protocol well‐established in the animal field for ER Ca^2+^ assays (Ottolini et al., 2014), the steady‐state lumenal [Ca^2+^]_ER_ was subsequently restored, after extensive washing‐out of CPA in 100 µm EGTA, by administration of 1 mm CaCl_2_. The overall experimental protocol of [Ca^2+^]_ER_ depletion/refill is schematically depicted in Figure 3. The ER Ca^2+^ refilling step induced a rapid and sustained increase in [Ca^2+^]_ER_, mimicking that commonly observed in the animal ER (Ottolini et al., 2014). The application of higher CaCl_2_ concentrations (2, 5, 10 mm) did not result in increased basal ER Ca^2+^ levels (expressed as luminescence/total residual luminescence at that moment, *L*/*L*_max_), ruling out the possibility that the reached [Ca^2+^] steady‐state was dependent on limited external Ca^2+^ supply (Figure S8).

### Monitoring ER Ca^2+^ signals in response to environmental cues and comparison with cytosolic and chloroplast Ca^2+^ signatures

**Figure 4:**
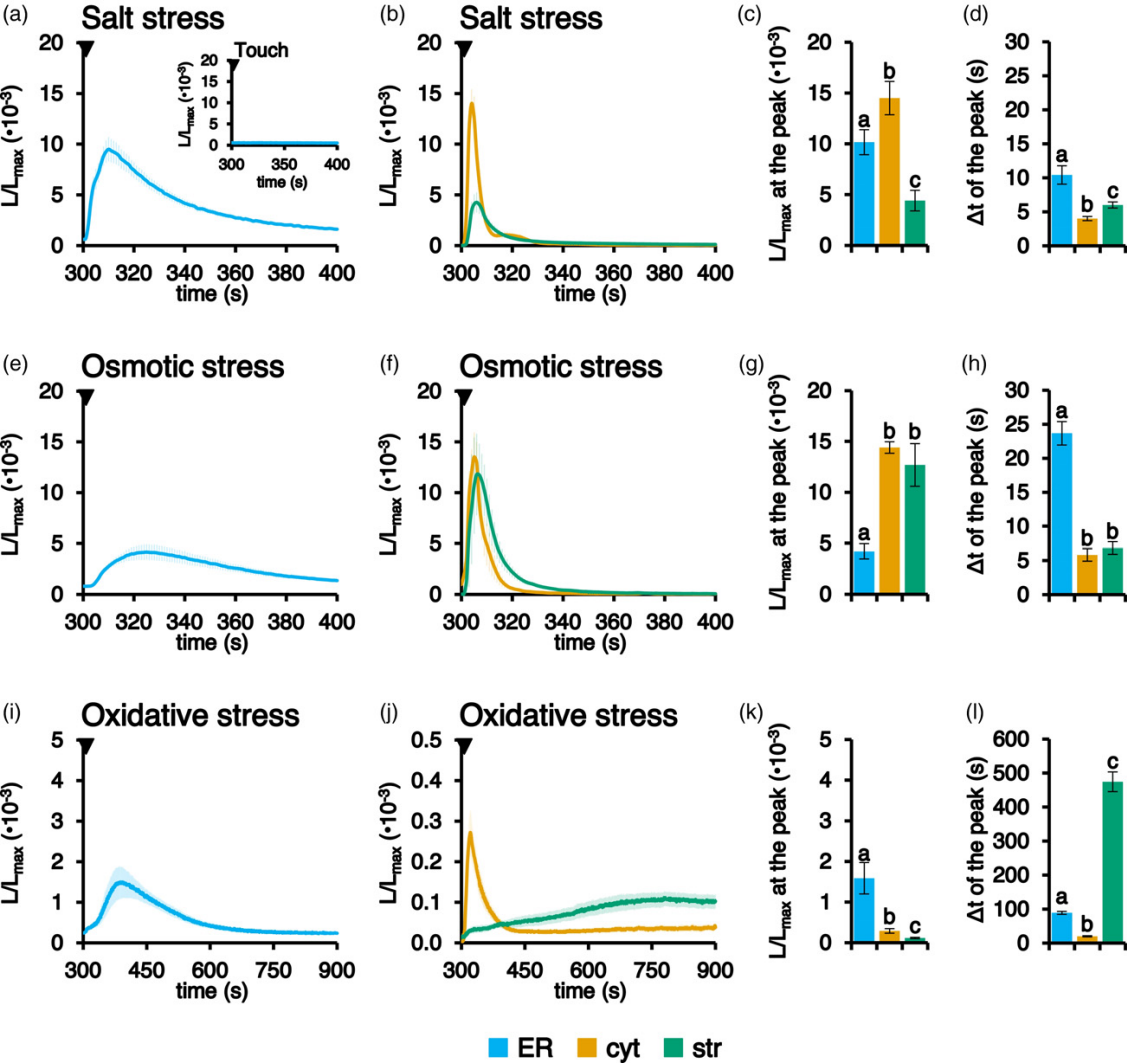
Comparison between [Ca^2+^] dynamics in the ER, cytosol and chloroplasts in response to environmental stimuli. Ca^2+^ measurements were performed in Arabidopsis seedlings stably expressing aequorin in the ER (light blue trace), cytosol (cyt, orange trace) or chloroplast stroma (str, green trace). Seedlings were challenged with different abiotic stresses: (a–d) 300 mm NaCl; (e–h) 600 mm mannitol; and (i–l) 10 mm H_2_O_2_. The time scale starts at 300 sec, the time point of stimulus addition (black arrowhead), after the [Ca^2+^]_ER_ refilling protocol. The inset in (a) shows a touch control (injection of an equal volume of H_2_O). Data are the mean (solid lines) ± SE (shading) of ≥ 6 different seedlings derived from three independent growth replicates. Statistical analyses of *L*/*L*_max_ at the peak (c, g, k) and delay of the peak after the stimulus injection (d, h, l) are shown. Bars labelled with different letters differ significantly (*P* < 0.05, Student’s *t* test).

To test the potential involvement of the ER in intracellular Ca^2+^ signalling evoked by environmental cues, fl2‐AEQmut Arabidopsis seedlings were challenged with different abiotic stimuli (i.e. touch, salt, osmotic, oxidative stresses). Application of a touch stimulus (injection of an equal volume of H_2_O) did not affect resting [Ca^2+^]_ER_ levels (Figure 4a, insert), whereas the application of 300 mm NaCl (mimicking a salt stress) led to a rapid (after 10.4 ± 1.4 sec) and transient, approximately 10‐fold increase in [Ca^2+^]_ER_ (Figure 4a,c,d). When seedlings were challenged with 600 mm mannitol to simulate an osmotic stress, an ER Ca^2+^ transient characterized by a comparatively halved‐peak was recorded after 23.7 ± 1.7 sec (Figure 4e,g,h), whereas an oxidative stress (10 mm H_2_O_2_) was found to induce a less pronounced and much slower Ca^2+^ elevation (after 89.0 ± 4.3 sec) (Figure 4i,k,l). Interestingly, none of the tested stimuli triggered plant ER Ca^2+^ release, but did evoke transient [Ca^2+^]_ER_ increases, characterized by stimulus‐specific dynamics. This is a striking difference from that commonly observed in animal cells, where the ER plays a predominant role as stimulus‐releasable Ca^2+^ store.

Ca^2+^ assays were also carried out in Arabidopsis seedlings stably expressing aequorin in either the cytosol or chloroplast stroma (Mehlmer et al., 2012; Sello et al., 2018) and the Ca^2+^ traces recorded in the respective intracellular compartments were compared with those obtained in the ER in response to the same abiotic stimuli (Figure 4b,f,j). In all considered cases, ER Ca^2+^ transients temporally followed cytosolic Ca^2+^ changes (Figure 4d,h,l). In particular, the peak of the ER Ca^2+^ response evoked by salt stress appeared to be only slightly delayed (6.4 ± 1.4 sec) compared to the cytosolic one (Figure 4a–d), whereas [Ca^2+^]_ER_ elevations triggered by osmotic stress (Figure 4e–h) and oxidative stress (Figure 4i–l) showed a peak occurring 17.8 ± 1.7 and 69.0 ± 4.3 sec after the cytosolic one, respectively. These data make it unlikely that the ER is involved in the generation of the cytosolic Ca^2+^ signals triggered by the above‐mentioned stimuli, but rather has a role in their dissipation. By contrast, Ca^2+^ assays demonstrated that there was no univocal temporal correlation between chloroplast and ER Ca^2+^ transients. Indeed, depending on the nature of the stimulus, the peaks of chloroplast Ca^2+^ transients occurred either before (4.4 ± 1.4 sec in the case of salt stress and 16.8 ± 1.7 sec in the case of osmotic stress) or after (385.5 ± 29.0 sec, oxidative stress) the ER Ca^2+^ peaks (Figure 4d,h,l).

[Ca^2+^]_ER_ dynamics were also monitored in response to biotic stimuli. The flg22 peptide (1 μm), derived from bacterial flagellin, did not evoke any evident [Ca^2+^]_ER_ change, at least in the observation time frame of 700 sec (Figure S9a). Likewise, when short chain chito‐oligosaccharides (COs with a degree of polymerization 2‐5, 1 μg ml^−1^) were applied to mimic the symbiotic signal released by arbuscular mycorrhizal (AM) fungi during the establishment of AM symbiosis with host plants (Volpe et al., 2020), the [Ca^2+^]_ER_ trace was almost superimposable to the trace obtained in the absence of COs application (control) (Figure S9b). On the other hand, a mild [Ca^2+^]_ER_ increase was recorded in response to oligogalacturonides (OGs with a degree of polymerization 10–15, 20 μg ml^−1^), comprising pectic fragments of the plant cell wall originating after pathogen attack (Figure S9c).

### Pharmacological approaches to investigate the origin of ER Ca^2+^ fluxes

**Figure 5:**
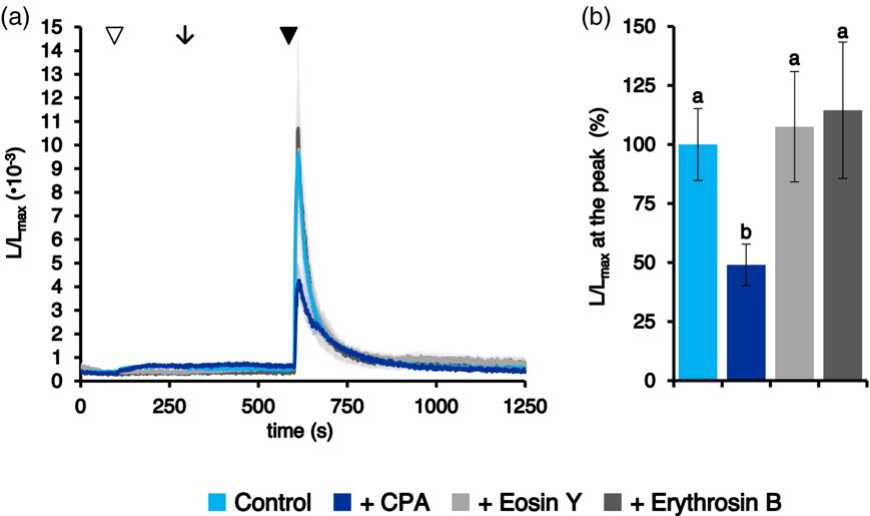
Pharmacological approach to the analysis of salt stress‐induced ER Ca^2+^ fluxes. Ca^2+^ assays were conducted in Arabidopsis seedlings stably expressing the aequorin chimera targeted to the ER. (a) After the administration of 1 mm CaCl_2_ (at 100 sec, white arrowhead) to restore the steady‐state [Ca^2+^]_ER_, seedlings were incubated (at 300 sec, black arrow) with H_2_O (control, light blue trace) or with different inhibitors of Ca^2+^‐ATPases: 50 μm CPA (dark blue trace), 1 μm eosin Y (light grey trace) and 1 μm erythrosin B (dark grey trace). At 600 sec (black arrowhead), all samples were challenged with 300 mm NaCl. Data are the mean (solid lines) ± SE (shading) of six different seedlings derived from three independent growth replicates. (b) Statistical analyses of *L*/*L*_max_ at the peak. Bars labelled with different letters differ significantly (*P* < 0.05, Student’s *t* test).

As a result of the expected significant differences between resting levels of [Ca^2+^] in the cytosol and in the ER, the participation of active Ca^2+^ transporters located at ER membranes in the observed stimulus‐induced [Ca^2+^]_ER_ increases seemed likely. To obtain insights into putative ER membrane‐localized Ca^2+^ transporters responsible for the observed ER Ca^2+^ increases, a pharmacological approach based on the use of specific inhibitors of plant Ca^2+^‐ATPases (Bonza and De Michelis, 2011; De Vriese et al., 2018; García Bossi et al., 2020) was applied. Ca^2+^ measurements were carried out in fl2‐AEQmut seedlings by pre‐treating them for 5 min with either the specific ECAs inhibitor CPA (50 μm) or the ACAs (Autoinhibited Ca^2+^‐ATPases) blockers eosin Y (1 μm) and erythrosin B (1 μm) before stimulus application (a salt stress). In line with the successful use of CPA in the coelenterazine reconstitution protocol, 51.0 ± 8.8% inhibition of the stimulus‐induced ER Ca^2+^ increase evoked by 300 mm NaCl was observed, whereas eosin Y and erythrosine B did not induce any significant change in the [Ca^2+^] elevation with respect to the control (Figure 5). These data indicate the main involvement of the CPA‐inhibited, ER‐type Ca^2+^‐ATPase ECA1 in the observed salt stress‐induced ER Ca^2+^ uptake; in contrast, the contribution of ER‐located ACAs appears negligible.

**Figure 6:**
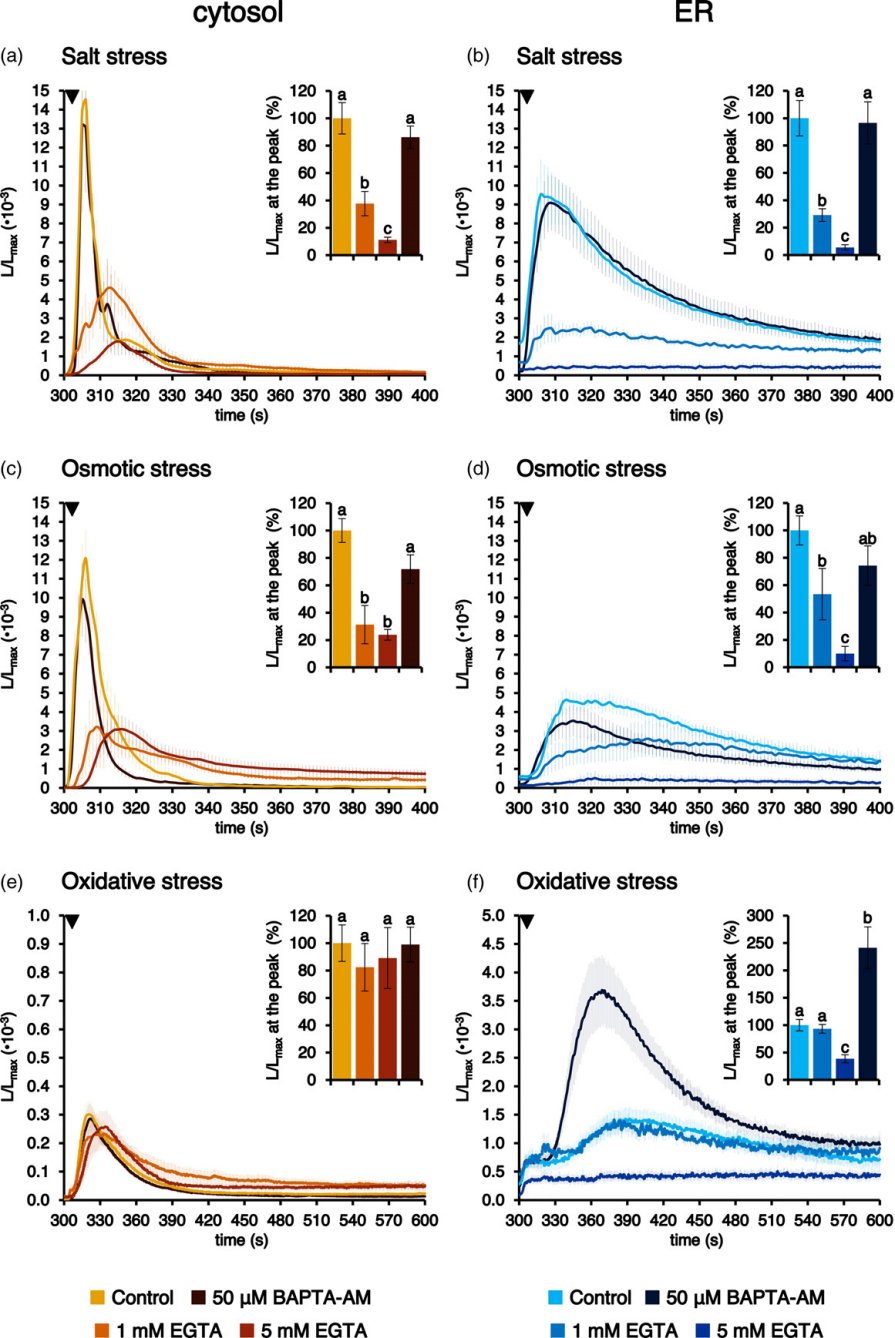
Effect of pre‐treatment with Ca^2+^ chelators on abiotic stresses‐triggered [Ca^2+^]_cyt_ and [Ca^2+^]_ER_ transients. Ca^2+^ assays were conducted in Arabidopsis seedlings stably expressing aequorin in the cytosol (a, c, e) or in the ER (b, d, f). Seedlings were incubated under control conditions (light traces) or pre‐treated with either 1 mm EGTA for 10 min (intermediate‐shade traces), 5 mm EGTA for 10 min (dark traces) or 50 μm BAPTA‐AM for 1 h (black traces), before challenge with different abiotic stresses (300 sec, black arrowhead): (a, b) 300 mm NaCl; (c, d) 600 mm mannitol; and (e, f) 10 mm H_2_O_2_. Data are the mean (solid lines) ± SE (shading) of ≥ 6 different seedlings derived from three independent growth replicates. Insets show statistical analyses of *L*/*L*_max_ at the peak. Bars labelled with different letters differ significantly (*P* < 0.05, Student’s *t* test).

To functionally link cytosolic and ER [Ca^2+^] elevations, Arabidopsis seedlings stably expressing aequorin in the cytosol or in the ER were pre‐treated with different concentrations of the extracellular Ca^2+^ chelator EGTA. Indeed, the extracellular milieu often represents a major Ca^2+^ source in a wide variety of Ca^2+^‐mediated signal transduction pathways (Costa et al., 2018; Resentini et al., 2021a). Pre‐treatment with 1 or 5 mm EGTA caused a reduction in the magnitude of [Ca^2+^]_cyt_ evoked in response of salt stress of 62.3 ± 8.9 and 88.8 ± 2.0%, respectively (Figure 6a). Similarly, in the case of osmotic stress, both doses of EGTA resulted in a significant inhibition of the stimulus‐evoked [Ca^2+^]_cyt_ (68.8 ± 14.0 and 76.2 ± 3.9%, respectively) (Figure 6c), whereas [Ca^2+^]_cyt_ changes in response to oxidative stress remained almost unaltered (Figure 6e).

Ca^2+^ responses in the ER were likewise highly reduced by EGTA pre‐treatment. In particular, 1 mm EGTA caused 70.8 ± 4.6 and 46.6 ± 18.8% inhibition of salt stress‐ and osmotic stress‐induced [Ca^2+^]_ER_ increases, respectively; an even higher inhibition (94.4 ± 2.0 and 90.0 ± 5.3%) was determined by 5 mm EGTA (Figure 6b,d). On the other hand, pre‐treatment of seedlings with 1 mm EGTA did not significantly reduce the magnitude of the ER Ca^2+^ transient in response to an oxidative stress and 5 mm EGTA caused a significant reduction (61.5 ± 7.3%) in the ER Ca^2+^ uptake, although this was less dramatic than that recorded for the other two abiotic stimuli (Figure 6f). Taken together, these data confirm the existence of both a temporal and a causal link between the Ca^2+^ transients in the cytosol and the ER. Pre‐treatment with the cell‐permeant Ca^2+^ chelator BAPTA‐AM (50 µm) was not found to effectively block either the cytosolic or ER Ca^2+^ rises (Figure 6a–e) in response to most of the tested abiotic stresses, with the sole exception of the oxidative stress‐induced [Ca^2+^]_ER_ transient, for which the magnitude was greatly increased (Figure 6f).

### Calibration of the aequorin‐based luminescence into [Ca^2+^]_ER_ values

**Figure 7:**
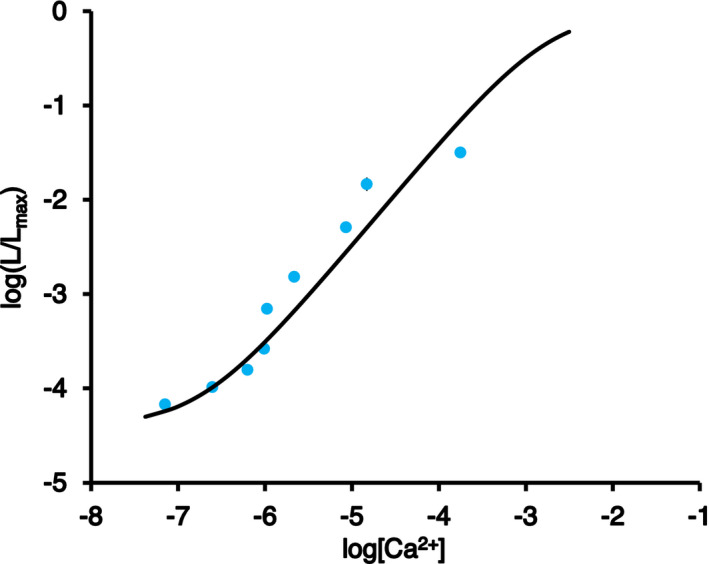
Determination of the [Ca^2+^] calibration curve for the plant ER‐targeted fl2‐AEQmut probe. Reconstituted protein crude extracts were obtained from Arabidopsis seedlings stably transformed with fl2‐AEQmut. Luminescence emitted at 22°C (L) upon injection of 200 µl of different Ca^2+^ concentrations to 50 µl of reconstituted extracts was measured. At the end of each experiment, the total residual luminescence (*L*_max_) was collected by allowing full consumption of the probe via the addition of 50 μl of 1 m CaCl_2_. The ratio *L*/*L*_max_ was plotted against actual [Ca^2+^]_free_ corresponding to the applied [CaCl_2_], which was measured under the same conditions using the fluorescent calcium indicator Calcium Green™‐5N. Data are the mean ± SE of three different biological samples, each including three technical replicates. The continuous curve corresponds to the best fit of the experimental data, as described in the Experimental Procedures.

To convert aequorin‐based luminescence data into [Ca^2+^]_ER_ values, an *in vitro* calibration curve was determined using cell lysates from fl2‐AEQmut transgenic lines. The relationship between the relative luminescence data (*L*/*L*_max_) and the corresponding free [Ca^2+^] was determined and plotted (Figure 7). The calibration curve represents the best fit for the experimental data, as described in Brini et al. (1995). The aequorin probe tethered to the plant ER membrane exhibited a high dynamic range, rendering it able to measure [Ca^2+^] ranging from the micromolar to submillimolar range. This Ca^2+^ response curve can be used to calibrate fl2‐AEQmut luminescence into [Ca^2+^]_ER_ values. Based on this curve, the steady‐state [Ca^2+^] in the ER was calculated to be in the low micromolar range (approximately 5 µm) (Figure 5), whereas it increased up to 10‐fold higher levels (approximately 50 µm) in response to the tested abiotic stimuli (Figures 4 and 5). These data highlight fundamental differences in basal [Ca^2+^]_ER_ and Ca^2+^ handling by the plant ER compared to the animal counterpart.

## DISCUSSION

Despite recent advances in the understanding of the mechanisms underlying ER architecture and dynamics in plant cells (Pain et al., 2019; Stefano and Brandizzi, 2018), knowledge of the Ca^2+^ handling properties of the plant ER has significantly lagged behind. This is partially because of the predominant role traditionally attributed to the vacuole as the main intracellular Ca^2+^ store of the plant cell (Peiter, 2011), which, for a long time, has overshadowed the potential contribution of other organelles, such as the ER, to intracellular Ca^2+^ homeostasis and signalling. The increasing availability of genetically encoded Ca^2+^ indicators (GECIs) specifically targeted to the ER has renewed interest in better understanding the role of the ER in Ca^2+^‐mediated signal transduction pathways. The targeting of fluorescent GECIs, i.e. the FRET‐based Ca^2+^ indicator cameleon (Bonza et al., 2013; Iwano et al., 2009) and, more recently, the single fluorescent protein‐based Ca^2+^ biosensor GCaMP (Luo et al., 2020; Resentini et al., 2021b) to the plant ER lumen has allowed excellent visualization of [Ca^2+^]_ER_ changes in response to different stimuli. In particular, the report of a long‐distance ER Ca^2+^ wave in response to wounding has opened an exciting new scenario that foresees the potential involvement of the ER in plant systemic signalling (Resentini et al., 2021b).

Although GFP‐based Ca^2+^ reporters are ideal for Ca^2+^ imaging, the Ca^2+^‐sensitive bioluminescent protein aequorin still remains the most suitable tool for accurately monitoring Ca^2+^ handling in a dynamic range of concentration values (Alonso et al., 2017; Costa et al., 2018; Grenzi et al., 2021; Greotti and De Stefani, 2020; Ottolini et al., 2014; Pérez Koldenkova and Nagai, 2013).

In the present study, an aequorin chimera, tethered to the ER membrane and facing the ER lumen, provided the means to quantify changes in [Ca^2+^]_ER_ during signal transduction, highlighting the role of plant ER in shaping intracellular Ca^2+^ signals. The novel ER‐targeted aequorin‐based Ca^2+^ sensor was accomplished by fusing the non‐cleavable ER signal peptide (fl2) of a maize mutant isolated in the mid‐to‐late 1990s (Coleman et al., 1995; Gillikin et al., 1997) to a point‐mutated, low‐Ca^2+^‐affinity, aequorin variant (AEQmut), commonly employed for [Ca^2+^]_ER_ measurements in the animal field (Ottolini et al., 2014).

Confocal and electron microscopy analyses confirmed the correct targeting of fl2‐fused YFP and AEQmut probes to ER membranes in transiently and stably‐transformed Arabidopsis plants. After checking the proper functionality of the ER‐targeted Ca^2+^ probe by *in vitro* reconstitution assays, the fl2‐AEQmut *in vivo* reconstitution procedure was carried out in agreement with a well‐established procedure commonly employed for ER Ca^2+^ signalling studies in mammalian cells, which involves [Ca^2+^]_ER_ depletion before aequorin reconstitution, followed by [Ca^2+^]_ER_ refilling (Brini, 2008; Ottolini et al., 2014). The calibration of the luminescence signal into [Ca^2+^]_ER_ values using an *ad hoc* Ca^2+^ response curve provided values for the basal Ca^2+^ level in the plant ER in the low micromolar range (approximately 5 µm). Although [Ca^2+^] in the plant ER was found to be approximately 10 to 100 times lower than that generally reported in the ER of animal cells (i.e. from 50 to 500 µm) (Coe and Michalak, 2009), this value is approximately 50 times higher than [Ca^2+^] in the cytosol (approximately 100 nm), rendering this intracellular compartment the second main intracellular Ca^2+^ store of the plant cell, after the vacuole (Peiter, 2011).

In special plant systems, such as the pollen tube, values of 100 to 500 µm were previously estimated by cameleon‐based studies (Iwano et al., 2009). Higher [Ca^2+^]_ER_ values than those reported in the present study were also indirectly inferred in previous studies, in which the fluorescent Ca^2+^ indicators CRT‐D4ER, R‐CEPIA1*er* and ER‐GCaMP6‐210 (characterized by *in vitro K*_d_ for Ca^2+^ in the high micromolar range) were used to visualize ER Ca^2+^ signals in Arabidopsis (Bonza et al., 2013; Luo et al., 2020; Resentini et al., 2021b). However, it must be considered that aequorin‐based probes do not allow measurements with cellular resolution, providing an average estimation of Ca^2+^ levels across the whole sample. Therefore, it is possible that the range of [Ca^2+^]_ER_ that we measured at steady‐state and during signal transduction in our experimental set up may refer to specific cell subpopulations within the plant. Further investigations are needed to evaluate the potential occurrence of heterogeneous distributions of [Ca^2+^]_ER_ in different plant tissues and organs.

Monitoring ER Ca^2+^ dynamics in response to several environmental stresses provided evidence for rapid and transient [Ca^2+^]_ER_ increases (up to 10‐fold the resting value) in response to salt, osmotic and oxidative stresses. Notably, the [Ca^2+^] response curve of fl2‐AEQmut exhibits a linear relationship between 10^−6^ and 10^−4^ m. This is in good agreement with the range of physiological [Ca^2+^]_ER_ obtained in our experiments, providing evidence for the reliability of the [Ca^2+^]_ER_ measurements performed with this aequorin chimera. Interestingly, no changes in [Ca^2+^]_ER_ were observed in response to bacterial flg22, whereas OGs were found to trigger a modest, but detectable, [Ca^2+^]_ER_ elevation. These data suggest a differential contribution of the ER as the Ca^2+^ store responsible for the modulation of cytosolic Ca^2+^ fluxes evoked by microbe‐ and damage‐associated molecular patterns (Choi and Klessig, 2016). On the other hand, the lack of a ER‐mediated Ca^2+^ response to short chain chito‐oligosaccharides (COs) was expected because COs have been demonstrated to represent a fungal symbiotic signal for the arbuscular mycorrhizal (AM) symbiosis that is established between AM fungi and most land plants, but not Arabidopsis (Genre et al., 2013).

The spectrum of Ca^2+^ signalling events in which the plant ER may be involved as a Ca^2+^ storage compartment from where the ion can be mobilized should be more widely investigated in the future. Indeed, a slow decline in [Ca^2+^]_ER_, generating a [Ca^2+^]_cyt_ elevation in the Arabidopsis root tip upon hydrostimulation has been reported (Shkolnik et al., 2018). Moreover, the cholinergic agonist carbachol has been shown to activate Ca^2+^ release from the ER in Arabidopsis, although the physiological meaning of this observation remains to be established (Luo et al., 2020).

Pharmacological approaches using classical inhibitors of plant Ca^2+^‐ATPases (CPA, eosin Y, erythrosin B) (De Vriese et al., 2018) demonstrated the main involvement of the CPA‐sensitive ER‐type Ca^2+^‐ATPase ECA1 in ER Ca^2+^ uptake, in agreement with previous studies (Bonza et al., 2013; Resentini et al., 2021b; Zuppini et al., 2004) and with its successful use for the [Ca^2+^]_ER_ depletion before aequorin reconstitution. Nevertheless, the participation of the recently characterized, ER‐located, cation/Ca^2+^ exchanger CCX2 in the control of Ca^2+^ fluxes between the ER and the cytosol (Corso et al., 2018) cannot be ruled out, especially because the use of CPA did not completely abolish the ER [Ca^2+^] transient. Moreover, the potential contribution of the ER Ca^2+^ buffering protein calreticulin (Joshi et al., 2019; Mariani et al., 2003) in modulation of [Ca^2+^]_ER_ increases remains to be investigated further. Indeed, recent reports support the role of plant calreticulin in the overall cellular Ca^2+^ homeostasis (Su et al., 2019; Suwińska et al., 2017).

The comparison of cytosolic and ER Ca^2+^ traces highlighted a time delay in the generation of ER Ca^2+^ transients with respect to cytosolic ones in response to different environmental stimuli, confirming and extending previous observations carried out using the ER‐targeted cameleon variant CRT‐D4ER (Bonza et al., 2013), as well as the GCaMP variant R‐CEPIA1*er* (Luo et al., 2020) and ER‐GCaMP6‐210 (Resentini et al., 2021b). Experiments performed by pre‐treating Arabidopsis seedlings with the extracellular Ca^2+^ chelator EGTA strongly supported the causal link underlying the temporal delay between the observed [Ca^2+^]_cyt_ and [Ca^2+^]_ER_ changes. In particular, the abolishment of the [Ca^2+^]_cyt_ changes observed in response to a salt and osmotic stresses in the presence of the cell‐impermeant Ca^2+^ chelator EGTA was mirrored by a corresponding inhibition of the [Ca^2+^]_ER_. The slightly different scenario observed in response to oxidative stress may be a result of the differential involvement of the apoplast in Ca^2+^ uptake and/or Ca^2+^ release from intracellular storage compartments (such as the vacuole) in response to distinct environmental stimulations. Moreover, the progressive decrease in the stimulus‐triggered [Ca^2+^]_ER_ transients when Arabidopsis seedlings were pre‐treated with higher doses of EGTA suggests that the depletion of the extracellular Ca^2+^ pool gradually leads to an increasing Ca^2+^ leakage from the ER and affects its ability to take up Ca^2+^ released in the cytoplasm upon stimulus application. Pre‐treatment with BAPTA‐AM was found to be ineffective in the chelation of intracellular Ca^2+^ fluxes, possibly because of cleavage of AM groups by extracellular esterases in the apoplast (De Vriese et al., 2018). Notably, in the case of oxidative stress, BAPTA‐AM pre‐treatment caused a remarkable increase of the [Ca^2+^]_ER_ transient, suggesting a non‐specific effect possibly as a result of alterations of the ER redox environment (Margittai et al., 2015).

Experiments carried out in parallel in Arabidopsis seedlings stably expressing aequorin in the chloroplast stroma added an extra level of complexity, revealing the participation of these organelles in the fine‐tuning of cytosolic Ca^2+^ signals by mediating Ca^2+^ fluxes between the cytosol and the ER, or at a later stage. The precise chloroplast–ER interplay in terms of Ca^2+^ handling needs to be investigated further using organelle‐targeted Ca^2+^ reporters in combination with pharmacological strategies and genetic approaches, such as specific inhibitors of Ca^2+^ transporters/channels and knockout plants defective in organellar Ca^2+^ transport and Ca^2+^ buffering mechanisms. The combined use of complementary strategies to measure and image intracellular Ca^2+^ will further advance future studies aimed at unravelling Ca^2+^‐mediated communication networks among the plant ER and other organelles.

## EXPERIMENTAL PROCEDURES

### Molecular cloning and construction of expression plasmids

The nucleotide sequence encoding the uncleavable ER signal peptide fl2 (MATKILALLALLALLVSATNV) of the 24‐kDa α‐zein of the maize mutant *floury2* (Coleman et al., 1995; Gillikin et al., 1997) was fused to the cDNA of a mutated version of aequorin (AEQmut), endowed with a reduced Ca^2+^ affinity (Montero et al., 1995). The sequence encoding AEQmut was amplified by PCR using XbaI_fl2_aeq, encoding the 21 amino acids of fl2, as forward primer and Aeq*Sac*I as reverse primer (Table S1). After digestion with *Xba*I and *Sac*I, the amplicon (751 bp) was cloned into the 35SCaMV cassette (677 bp) of the plasmid p35SCaMV. The entire cassette 35SCaMV‐fl2‐AEQmut was then amplified to create additional restriction sites (*Not*I and *Xho*I) and moved into the binary vector pGreen 0029. To obtain the fl2‐YFP construct, the sequence encoding the YFP was amplified by PCR using XbaI_fl2_Yfp and Yfp_rev as primers (Table S1). The amplicon (808 bp) was digested with *Xba*I and *Sac*I and cloned into the 35S‐CaMV cassette. After digestion with *EcoR*V, the entire cassette was then moved into the binary vector pGreen 0029.

### **fl2‐YFP transient expression in** *N. benthamiana* **and Arabidopsis**

Transient expression of the fl2‐YFP construct for localization studies was performed in both *N. benthamiana* and *A. thaliana* (Arabidopsis) Col‐0 ecotype. Standard agroinfiltration procedures were applied to fully‐expanded leaves of 4‐week‐old *N. benthamiana* (Sparkes et al., 2006) and Arabidopsis (Lee and Yang, 2006). Protoplasts isolated from wild‐type Arabidopsis cell suspension cultures were transformed by polyethylene glycol as described by Yoo et al. (2007).

### Generation of transgenic Arabidopsis lines

Arabidopsis Col‐0 plants were transformed by the floral dip technique (Clough and Bent, 1998) with the pGreen 0029‐fl2‐AEQmut construct to generate multiple transgenic lines. The same approach was used also for the pGreen 0029‐fl2‐YFP construct. The seeds of the F_1_ generation were surface‐sterilized and screened on agarized (0.8% w/v) half‐strength MS medium, pH 5.5 containing 50 μg ml^−1^ kanamycin (seedlings were grown at 21°C under a 16:8 h light/dark cycle). Plants that survived were transferred into single pots and grown on soil so that F_2_ generation seeds could be collected separately: the progeny of each F_1_ seedling was subsequently screened for aequorin expression both at the RNA and protein levels.

### Analysis of aequorin expression

Leaves from each of the Arabidopsis fl2‐AEQmut independent transgenic lines were collected from the F_2_ generation (kanamycin‐resistant, 1‐month‐old plants) and flash‐frozen in liquid nitrogen. Total RNA was extracted using the RNeasy Plant Mini Kit (Qiagen, Hilden, Germany) and then reverse transcribed with SuperScript III (Thermo Fisher Scientific, Waltham, MA, USA) in accordance with the manufacturer’s instructions. Primers designed on the cDNA sequence of fl2‐AEQmut (Table S1) and on the coding sequence of actin, used as control, were used to analyse gene expression (Sello et al., 2018). Total protein extraction from leaves of transgenic Arabidopsis plants (1‐month‐old), SDS‐PAGE and immunoblot analyses were carried out as described previously (Zonin et al., 2011). A polyclonal anti‐aequorin antibody (Abcam, Cambridge, UK) was used at a 1:5000 dilution. A purified His‐tagged aequorin was used as a positive control (Moscatiello et al., 2014).

### Set up of cell suspension cultures from Arabidopsis transgenic lines

Seeds of Arabidopsis fl2‐AEQmut and fl2‐YFP lines (F_3_ generation) that revealed the highest probe expression were used to establish cell suspension cultures, as described recently (Cortese et al., 2021).

### Microscopy analyses

The abaxial epidermis of *N. benthamiana* leaves infiltrated with *Agrobacterium* harbouring the fl2‐YFP construct was imaged using a LSM 880 confocal microscope (Zeiss, Oberkochen, Germany) after 72 h. Confocal microscopy observations of Arabidopsis (after transient and stable transformation with the fl2‐YFP construct) were performed with a TCS SP5 II confocal laser scanning system (Leica, Wetzlar, Germany) mounted on a DMI6000 inverted microscope (Leica). Samples were excited with a 488 nm Argon laser (for YFP and chlorophyll), a 543 nm Helium/Neon laser (for ER‐Tracker Red; Thermo Fisher Scientific) and a 561 nm diode laser for RFP, whereas fluorescence emissions were collected at 505–540 nm for YFP, 580–610 nm for RFP, 680–720 nm for chlorophyll and 600–633 nm for ER‐Tracker Red. Immunofluorescence experiments were conducted on cell suspension cultures stably expressing fl2‐AEQmut, as described by Zonin et al. (2011). Labelling was carried out with the anti‐aequorin antibody, diluted 1:1000, followed by Alexa Fluor 594 donkey anti‐rabbit Ig (Thermo Fisher Scientific). Cells were observed under a DM5000 B fluorescence microscope (Leica), with excitation at 515/560 nm and emission above 580 nm, and images were acquired with a DFC425 C digital camera (Leica), using LAS software (Leica).

TEM analyses and immunogold labelling (dilution 1:500) of 2‐week‐old Arabidopsis transgenic plants were carried out as described previously (Sello et al., 2018). Observations were carried out with a Tecnai G^2^ transmission electron microscope (Field Electron and Ion Company, Hillsboro, ‎OR‎, USA) operating at 100 kV and equipped with an Osis Veleta camera (Olympus, Tokyo, Japan).

### Measurement of photosynthetic efficiency

Quenching analyses were performed on 2‐week‐old seedlings grown under sterility conditions on agarized medium [half‐strength MS medium supplemented with 1.5% (w/v) sucrose, 0.8% (w/v) agar, under a 16:8 h light/dark cycle at 21°C]. Seedlings were exposed to 16 h of light followed by 20 min of dark adaptation prior to subsequent analyses. Fluorescence was detected by PAM imaging using FluorCam7 (Photon Systems Instruments, Drásov, Czechia): each min for 10 min, a saturation pulse of 2000 µE m^−2^ sec^−1^ intensity was applied for 800 msec. For the first 5 min (L_1_–L_5_), the seedlings were exposed to an actinic light of 650 µE m^−2^ sec^−1^, whereas, for the last 5 min (D_1_–D_5_), the seedlings were kept in the dark. Maximum fluorescence, non‐photochemical quenching and PSII quantum yield were analyzed.

### *In* *vitro* reconstitution of apoaequorin to aequorin and determination of the fl2‐AEQmut [Ca^2+^] calibration curve

Protein crude extracts were obtained from Arabidopsis transgenic and wild‐type cell suspension cultures (4 days old) using as reconstitution buffer 150 mm Tris‐HCl, 10 mm EGTA, 0.8 mm phenylmethylsulfonyl fluoride, pH 8.0. Proteins were resuspended at 1 μg μl^−1^ in reconstitution buffer and incubated with 1 mm β‐mercaptoethanol and 5 μm coelenterazine (Prolume, Pinetop, AZ, USA) for 4 h at 4°C in the dark. Aequorin luminescence was detected from 50 μl of the *in vitro* aequorin reconstitution mixture and integrated for 200 sec after the addition of an equal volume of 100 mm CaCl_2_.

To determine the [Ca^2+^] calibration curve of the aequorin probe at 22°C, protein crude extracts were likewise obtained from both fl2‐AEQmut #6 and #10 transgenic lines using a similar buffer with a reduced EGTA content (1 mm) and reconstituted according to the same protocol with the sole exception of a 10‐fold concentration of β‐mercaptoethanol (10 mm). At the end of the reconstitution, proteins were further diluted to 0.5 μg μl^−1^ using an EGTA‐free equivalent buffer to lower the total EGTA concentration to 0.5 mm. Reconstituted extracts (50 μl per well, corresponding to 25 μg of total proteins) were placed in a 96‐well microplates (Corning Inc., Corning, NY, USA) to which a BackSeal (Perkin Elmer, Waltham, MA, USA) was applied: using an EnVision 2105 XCite multimode plate reader (Perkin Elmer), emitted luminescence at 22°C was recorded and integrated for 120 sec, following the administration at 3 sec of 200 μl of a variable [CaCl_2_] (ranging from 5.86 to 250 μm) – which further reduced the total EGTA concentration to 0.1 mm – and the addition at 60 sec of a discharge‐like solution (1 m CaCl_2_). The collected data were analyzed and expressed as *L*/*L*_max_ (i.e. the ratio between instantaneous emitted luminescence and total residual luminescence) for each applied [CaCl_2_].

To measure the actual free [Ca^2+^] corresponding to each applied [CaCl_2_] under these experimental conditions, 50 µl of reconstitution buffer (either without EGTA or containing 0.5 mm EGTA) was complemented with 1 µm Calcium Green™‐5N and 10 µm *N*,*N*,*N*′,*N*′‐tetrakis(2‐pyridinylmethyl)‐1,2‐ethanediamine and used instead of the reconstituted protein extracts. By following an equivalent protocol, fluorescence collected with a Green Fluo filter (excitation at 485/14 nm, emission at 535/30 nm), allowing the determination of the Calcium Green™‐5N affinity curve for Ca^2+^ (specific for these ionic strength, temperature and pH conditions), which was then used to calculate the exact free [Ca^2+^] plotted against *L*/*L*_max_ values. The calibration curve, representing the best fit for the experimental data, was obtained as described by Brini et al. (1995).

### *In vivo* **reconstitution of apoaequorin to aequorin**

Transgenic Arabidopsis seedlings (14 days old) (F_3_) were transferred in a microplate well and incubated in 600 μm EGTA solution supplemented with 5 μm coelenterazine. To set up an efficient *in vivo* reconstitution protocol, different procedures were experimented (wild‐type coelenterazine or coelenterazine *n*; incubation for 2, 4 and 8 h or overnight; pre‐treatment (10 min) with the ionophore A23187 (10 μm) or the ER Ca^2+^‐ATPase blocker CPA (50 μm) or both). Seedlings were floated on 1 ml H_2_O (six seedlings per microplate) and treated with A23187/CPA 10 min before the addition of coelenterazine. Afterwards, seedlings were extensively washed with 100 μm EGTA (10 to 20 ml per seedling) and allowed to recover for 10 min, before being singly transferred in the chamber (1 ml) of the luminometer (ET Enterprises Ltd, Uxbridge, UK) and subjected to challenge with stimuli and/or *in vivo* discharge (Figure S9). Cell viability was determined in cell suspension cultures derived from the Arabidopsis transgenic lines by the Evans Blue method (Baker and Mock, 1994).

### Aequorin‐based Ca^2+^ measurement assays

The best reconstitution protocol (pre‐treatment with 50 μm CPA + 2 h of incubation with wild‐type coelenterazine) was applied to all subsequent Ca^2+^ measurements carried out in the luminometer. Each experiment started with one Arabidopsis seedling incubated in 150 µl of 100 μm EGTA, to which an equal volume of a two‐fold (2 mm) CaCl_2_ solution was added after 100 sec to restore resting [Ca^2+^]_ER_. ER refill trials with higher [CaCl_2_] followed the same procedure but with appropriate [CaCl_2_] solutions. At 300 sec, 300 µl of a two‐fold concentrated stock solution for each tested stimulus was injected in the luminometer chamber. Oligogalacturonides (OGs with a degree of polymerization 10–15; Moscatiello et al., 2006), flg22 (GenScript, Piscataway, NJ, USA) and short chain chito‐oligosaccharides (COs with a degree of polymerization 2‐5; Zhengzhou Sigma Chemical Co. Ltd, Zhengzhou, China) were applied as biotic stimuli. All experiments were terminated by discharging the remaining aequorin pool with 400 µl of 1 m CaCl_2_, 30% (v/v) ethanol (Figure 3). For pharmacological studies, seedlings were pre‐treated with either 50 µm CPA, 1 µm eosin Y, 1 µm erytrosin B (Merck Life Science, Darmstadt, Germany), 5 min before stimulus application. Arabidopsis seedlings stably expressing YFP‐aequorin targeted to the cytosol (Cyt‐YA) or plastid stroma (Str‐YA) were also used in Ca^2+^ assays, as described previously (Sello et al., 2018). For experiments carried out with Ca^2+^ chelators, seedlings were pre‐treated either for 10 min with 1 or 5 mm EGTA or for 1 h with 50 µm BAPTA‐AM. Luminescence data were converted off‐line into [Ca^2+^]_ER_ values using a computer algorithm based on the previously determined Ca^2+^ response curve.

## AUTHOR CONTRIBUTIONS

EC and FP set up and carried out Ca^2+^ measurement assays. RM designed plasmids and performed gene expression analyses. LC and EC performed PAM analyses. LC and LF carried out plant transformation and confocal microscopy observations. BB performed TEM analyses. DDS and EC constructed the Ca^2+^ calibration curve. MB, LF, DDS, IS and UCV contributed to the discussion of the results and editing of the manuscript. LN conceived the research and designed the experiments. EC and LN analysed the data and wrote the article.

## CONFLICT OF INTERESTS

The authors declare no conflict of interest.

## Supporting information


**Figure S1**. Cloning strategy for the creation of the expression vector targeting the AEQmut probe to the plant ER and analysis of aequorin expression in Arabidopsis transgenic lines.

**Figure S2**. Phenotype, photosynthetic efficiency and ultrastructure of Arabidopsis transgenic lines stably expressing fl2‐AEQmut.

**Figure S3**. Confocal microscopy analyses demonstrate the ER localization of fl2‐YFP in Arabidopsis.

**Figure S4**. Immunofluorescence analyses of Arabidopsis cell suspension cultures stably expressing fl2‐AEQmut.

**Figure S5**. *In vitro* reconstitution assays in Arabidopsis fl2‐AEQmut transgenic lines.

**Figure S6**. *In vivo* reconstitution assays in Arabidopsis fl2‐AEQmut transgenic lines.

**Figure S7**. Effect of the fl2‐AEQmut reconstitution protocol on Arabidopsis cell viability.

**Figure S8**. Steady‐state [Ca^2+^]_ER_ is independent of the concentration of CaCl_2_ used in the refilling step.

**Figure S9**. Monitoring of [Ca^2+^]_ER_ dynamics in response to stimuli of biotic nature.

**Table S1**. List of primers used to target the fl2‐fused probes to the ER.

**Movie S1.** Time‐lapse confocal microscopy of a cortical sector of an Arabidopsis leaf epidermal cell stably expressing fl2‐YFP.

## Data Availability

All relevant data can be found within the manuscript or the supplementary material. All data and materials reported in this manuscript are available from the corresponding author upon reasonable request.
