# Supplementary figures and images for: Monitoring calcium handling by the plant endoplasmic reticulum with a low‐Ca2+‐affinity targeted aequorin reporter
Source: Plant J. 2021 Dec 11;109(4):1014–27. doi: 10.1111/tpj.15610 (PMC9299891; doi:10.1111/tpj.15610)

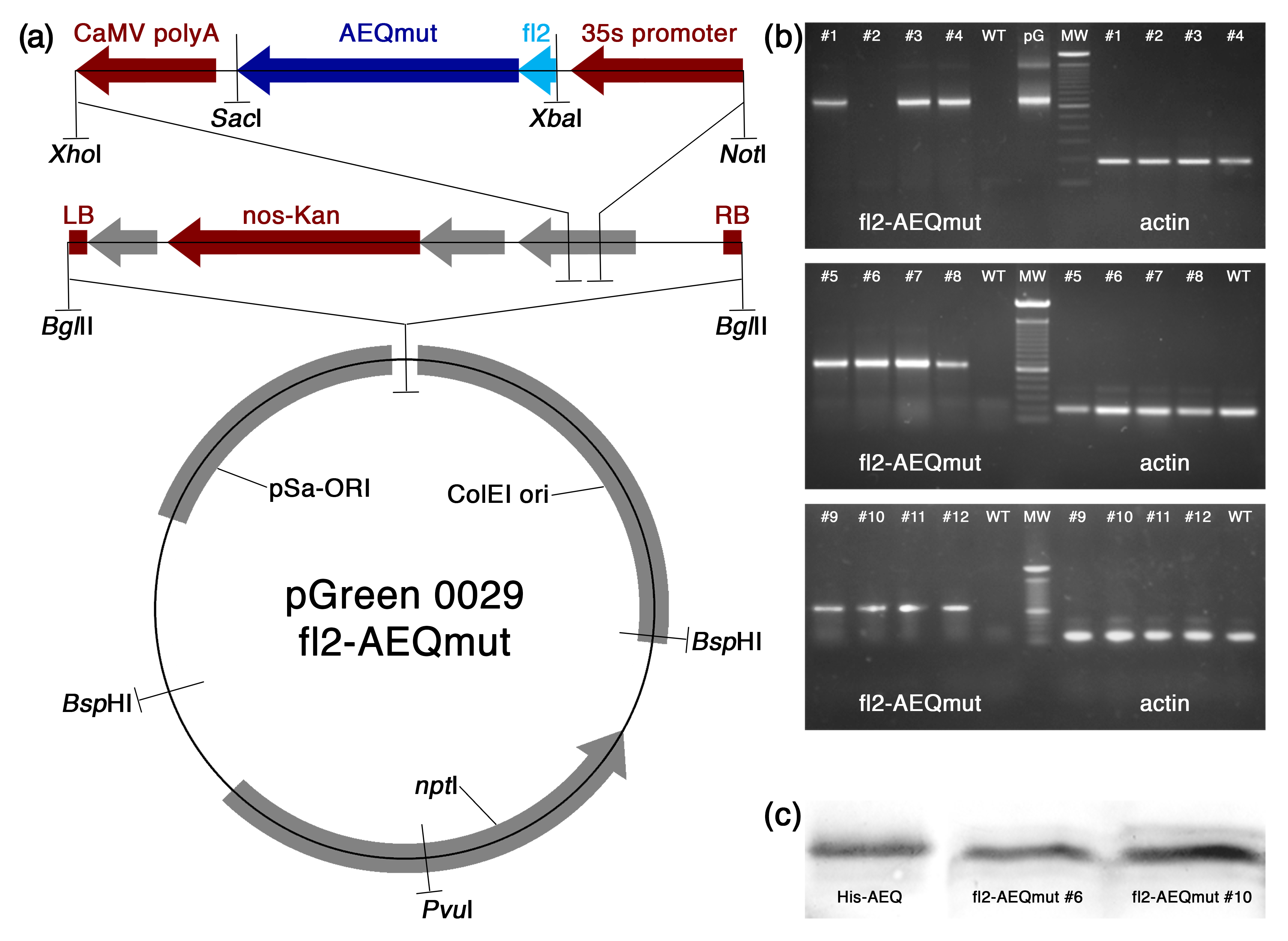

Supplement: Supplementary file 1 — Figure S1. Cloning strategy for the creation of the expression vector targeting the AEQmut probe to the plant ER and analysis of aequorin expression in Arabidopsis transgenic lines. [file TPJ-109-1014-s002.tif]

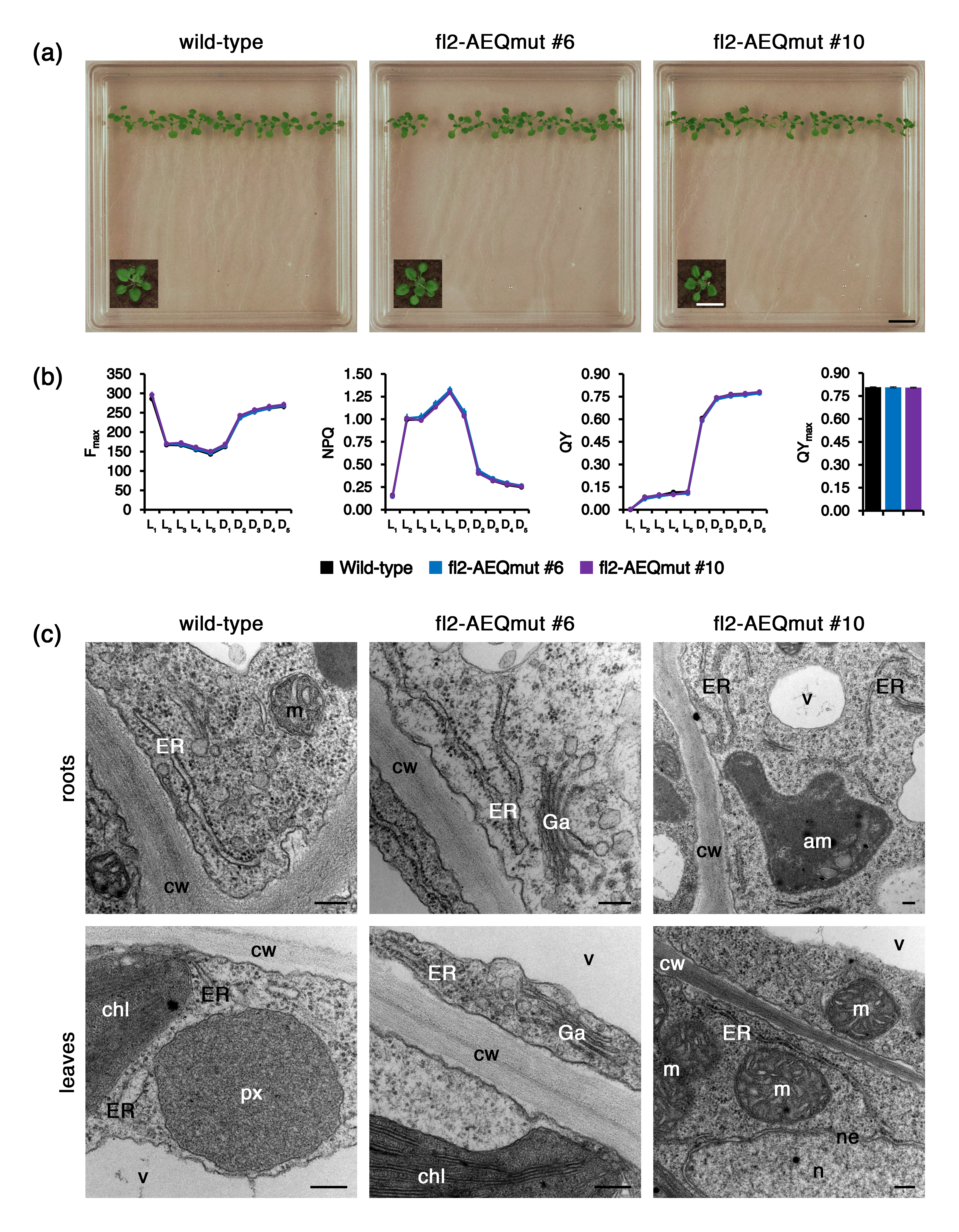

Supplement: Supplementary file 2 — Figure S2. Phenotype, photosynthetic efficiency and ultrastructure of Arabidopsis transgenic lines stably expressing fl2‐AEQmut. [file TPJ-109-1014-s007.tif]

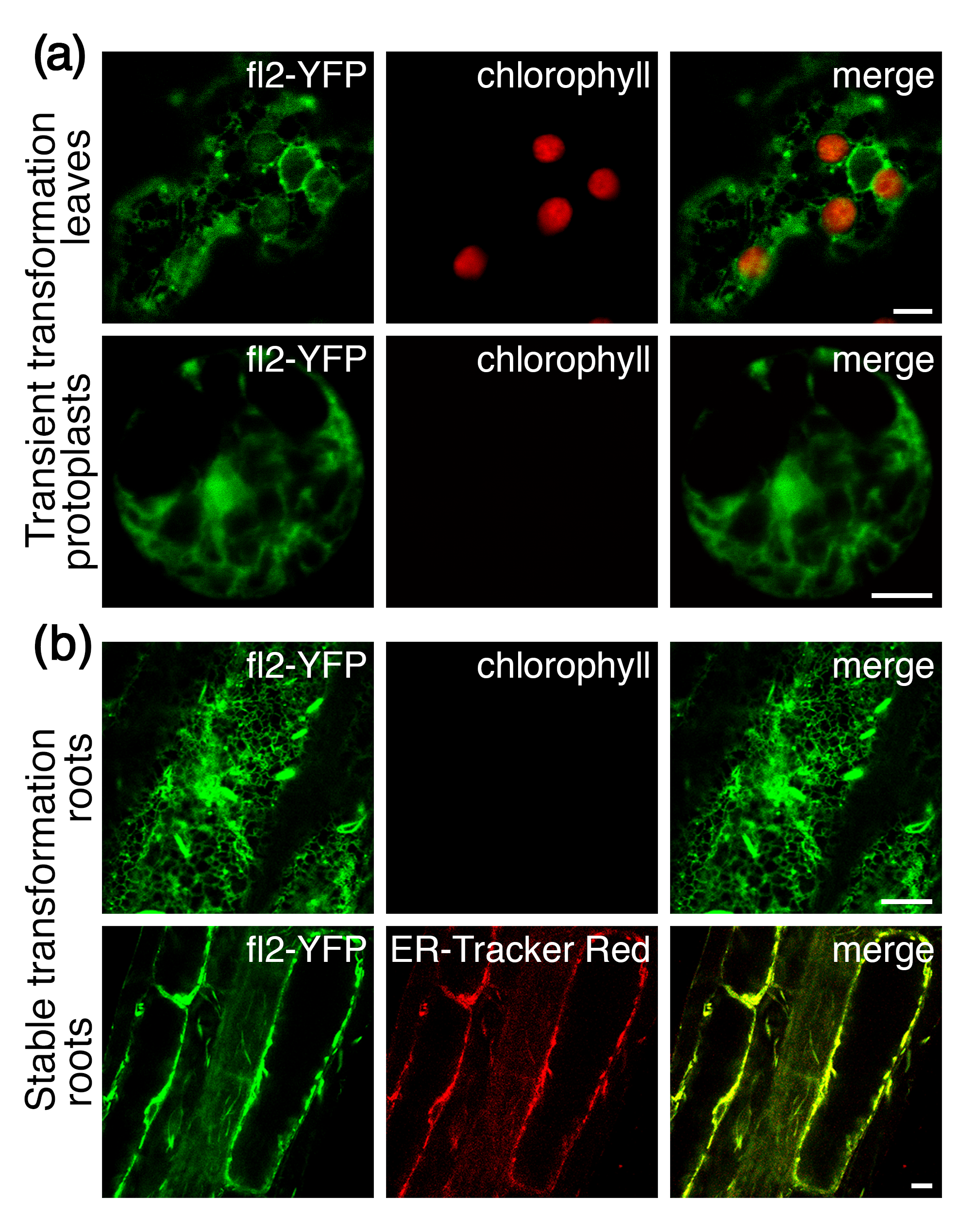

Supplement: Supplementary file 3 — Figure S3. Confocal microscopy analyses demonstrate the ER localization of fl2‐YFP in Arabidopsis. [file TPJ-109-1014-s001.tif]

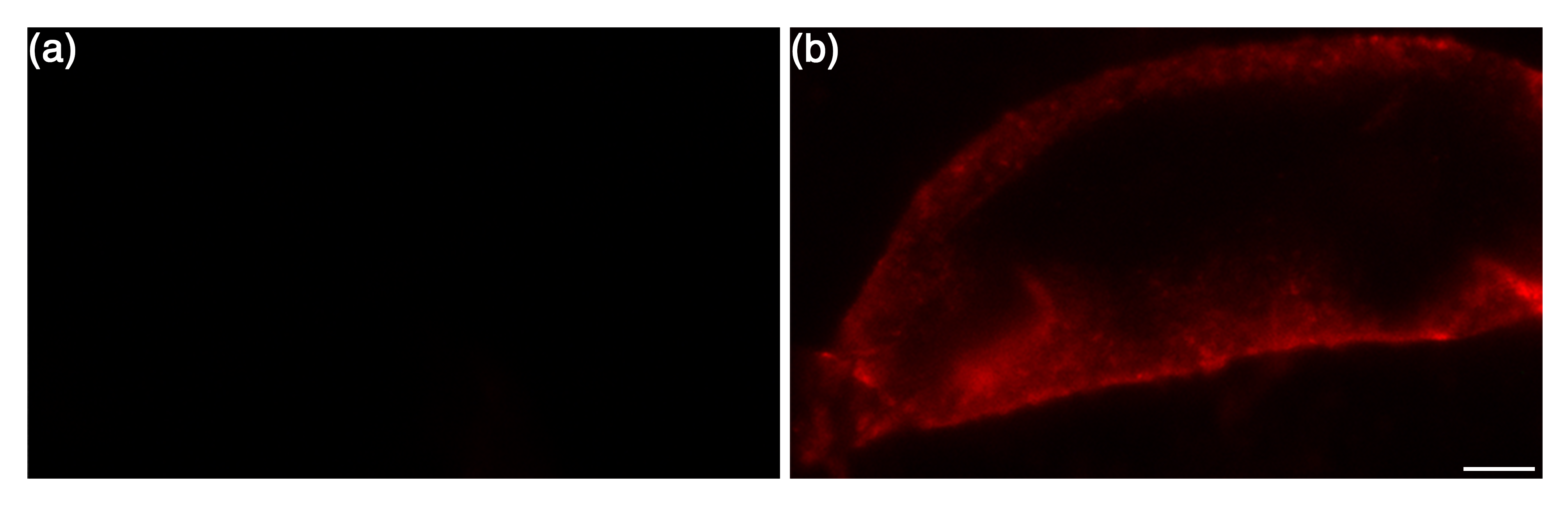

Supplement: Supplementary file 4 — Figure S4. Immunofluorescence analyses of Arabidopsis cell suspension cultures stably expressing fl2‐AEQmut. [file TPJ-109-1014-s003.tif]

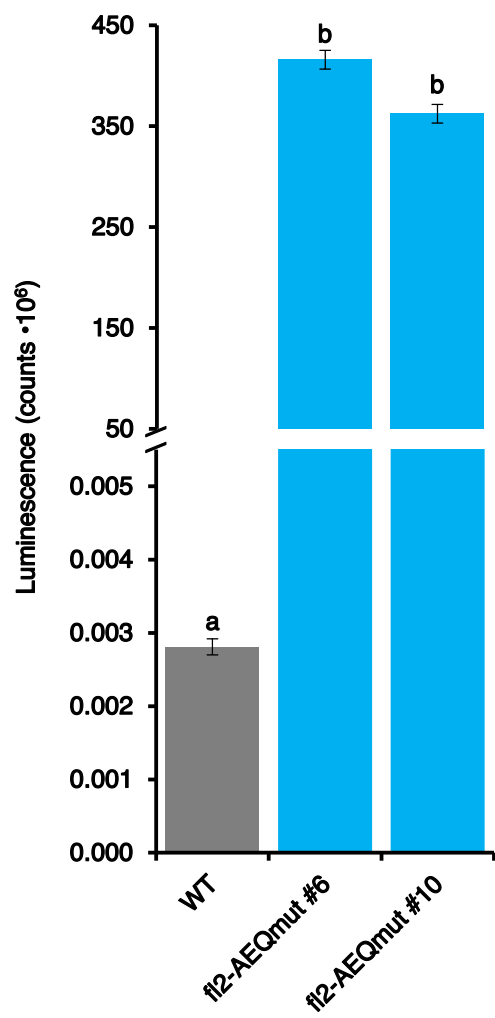

Supplement: Supplementary file 5 — Figure S5. In vitro reconstitution assays in Arabidopsis fl2‐AEQmut transgenic lines. [file TPJ-109-1014-s006.pdf]

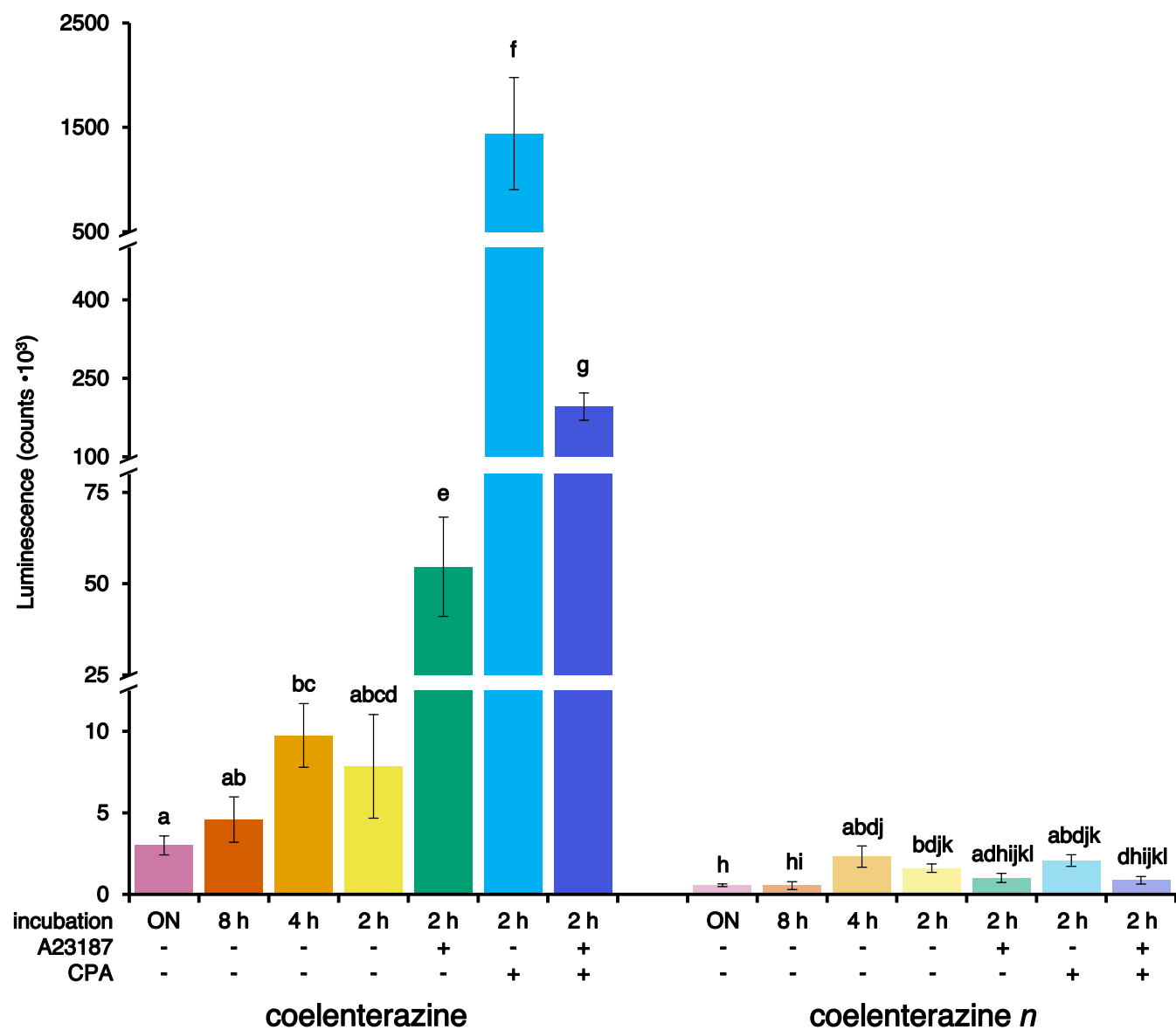

Supplement: Supplementary file 6 — Figure S6. In vivo reconstitution assays in Arabidopsis fl2‐AEQmut transgenic lines. [file TPJ-109-1014-s011.pdf]

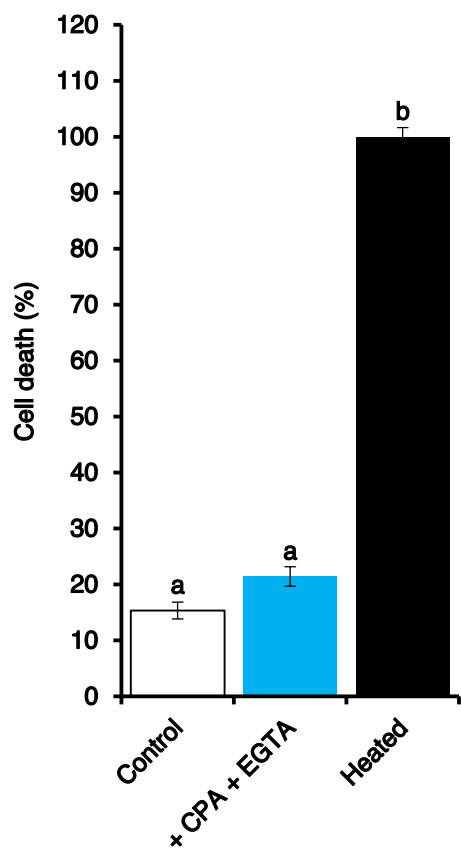

Supplement: Supplementary file 7 — Figure S7. Effect of the fl2‐AEQmut reconstitution protocol on Arabidopsis cell viability. [file TPJ-109-1014-s008.pdf]

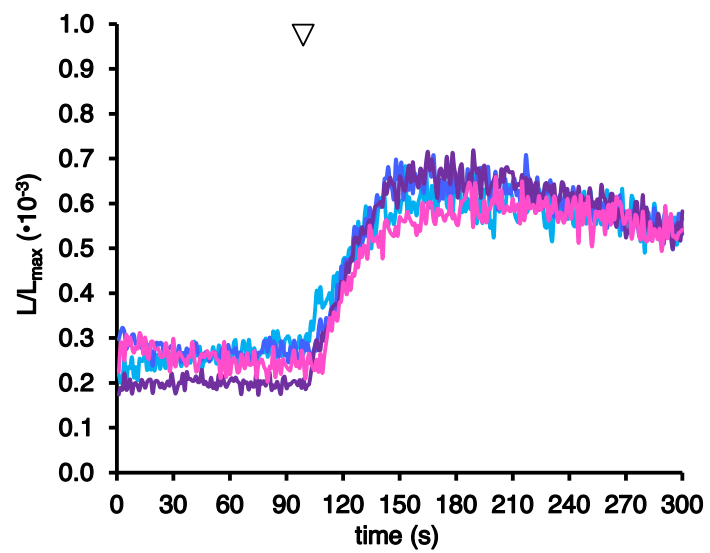

$\text{CaCl}_2$ : 1 mM 2 mM 5 mM 10 mM

Supplement: Supplementary file 8 — Figure S8. Steady‐state [Ca2+]ER is independent of the concentration of CaCl2 used in the refilling step. [file TPJ-109-1014-s010.pdf]

(a) Bacterial elicitor

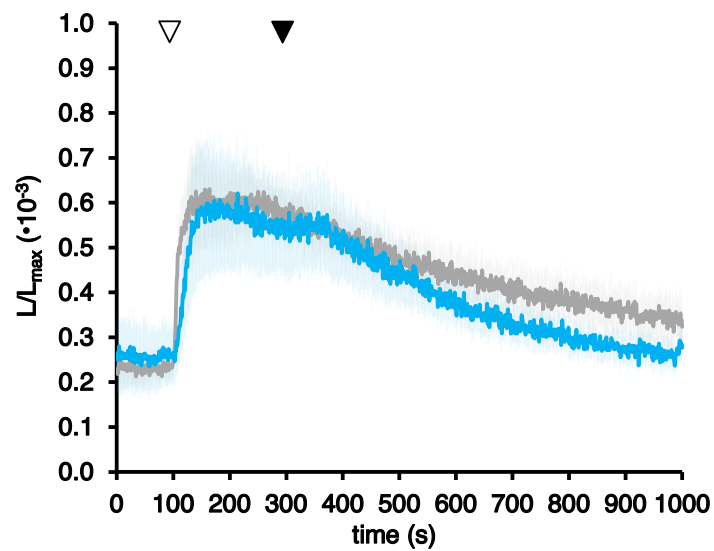

(b) Fungal symbiotic signal

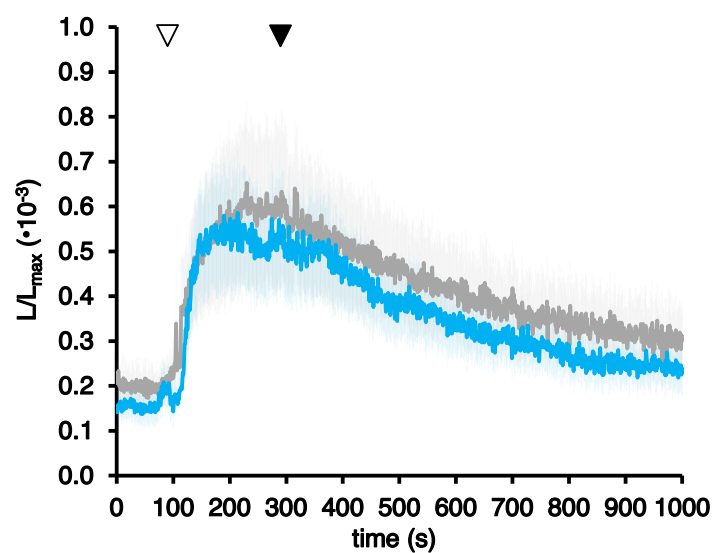

(c) Plant cell wall-derived elicitor

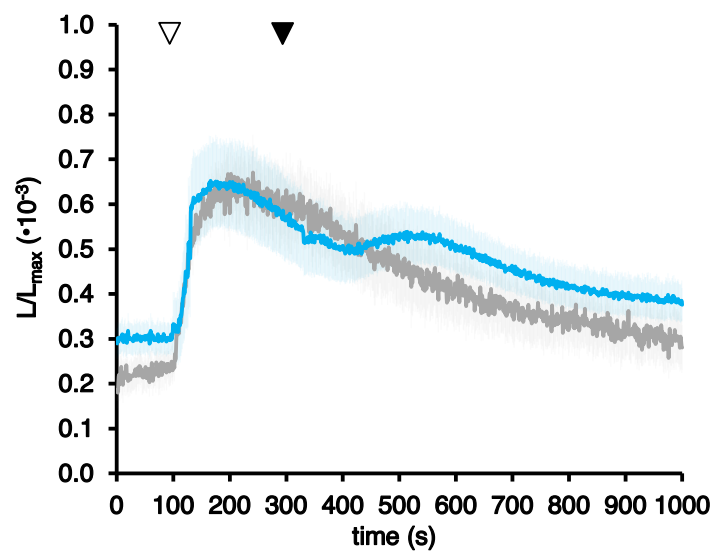

Supplement: Supplementary file 9 — Figure S9. Monitoring of [Ca2+]ER dynamics in response to stimuli of biotic nature. [file TPJ-109-1014-s005.pdf]
