# Supplementary material for: Monitoring calcium handling by the plant endoplasmic reticulum with a low‐Ca2+‐affinity targeted aequorin reporter
Source: Plant J. 2021 Dec 11;109(4):1014–27. doi: 10.1111/tpj.15610 (PMC9299891; doi:10.1111/tpj.15610)
Supplement: Supplementary file 10 — Table S1. List of primers used to target the fl2‐fused probes to the ER. [file TPJ-109-1014-s009.docx]

| **Name** | **5’-3’ Sequence** |
| --- | --- |
| **XbaI_fl2_aeq** | cttactctagaatggctaccaagatattagccctccttgcgcttcttgcccttttagtgagcgcaacaaatgtgaagctttatgatgttcctga |
| **AeqSacI** | tgatagagctcgaattcatcagtgttttat |
| **35S *Not*I_for** | atggcggccgcgatatcgtacccctactccaaaaat |
| **35S *Xho*I_rev** | catgctcgaggatatcgatctggattttagta |
| ***Xba*I_fl2_Yfp** | cttactctagaatggctaccaagatattagccctccttgcgcttcttgcccttttagtgagcgcaacaaatgtgggcagcaagggcgaggagct |
| **Yfp_rev** | tgatagagctcctagatcaccttgtacagc |
| ***Xba*I_fl2** | cttactctagaatggctaccaagatattag |
