# Supplementary material for: Monitoring calcium handling by the plant endoplasmic reticulum with a low‐Ca2+‐affinity targeted aequorin reporter
Source: Plant J. 2021 Dec 11;109(4):1014–27. doi: 10.1111/tpj.15610 (PMC9299891; doi:10.1111/tpj.15610)
Supplement: Supplementary file 12 [file TPJ-109-1014-s004.docx]

**Supporting Information Legends**

**Figure S1.** Cloning strategy for the creation of the expression vector targeting the AEQmut probe to the plant ER and analysis of aequorin expression in Arabidopsis transgenic lines. (a) The ER targeting sequence fl2 was cloned into a 35S-CaMV expression cassette in front of the low-affinity AEQmut probe by using the restriction enzymes *Xba*I and *Sac*I. The complete expression cassette was subsequently transferred into the binary vector pGreen 0029, harbouring kanamycin resistance, by using the restriction enzymes *Not*I and *Xho*I. (b) RT-PCR analyses of aequorin expression were performed on 12 independent F_2_ kanamycin-resistant lines; the wild-type (WT) line was used as negative control; the pGreen 0029 vector (pG) containing the 35SCaMV cassette with the fl2-AEQmut construct was used as positive control. Actin was used as a housekeeping gene. Among the 12 screened lines, only one (#2) turned out to be a false positive. (c) For immunoblot analyses, total protein extracts (50 μg) were separated by 12.5% SDS-PAGE, transferred to PVDF and incubated with an anti-aequorin antibody (1:5,000 diluted). Only the two lines fl2-AEQmut #6 and #10 showing the highest expression levels are shown. A His-tagged aequorin was used as positive control.

**Figure S2.** Phenotype, photosynthetic efficiency and ultrastructure of Arabidopsis transgenic lines stably expressing fl2-AEQmut. (a) Representative photographs of 2-week-old seedlings of fl2-AEQmut #6, fl2-AEQ #10 and wild-type lines. Inset, 4-week-old seedlings. (b) PAM imaging analyses of Arabidopsis seedlings. Data are the means ± SE of 20 seedlings derived from 6 independent growth replicates. (c) TEM observation of the ultrastructural organization of roots and leaves. am, amyloplast; cw, cell wall; ER, endoplasmic reticulum; Ga, Golgi apparatus; m, mitochondrion; n, nucleus; ne, nuclear envelope; px, peroxisome; v, vacuole. Bars, 1 cm (a) and 200 nm (c).

**Figure S3.** Confocal microscopy analyses demonstrate the ER localization of fl2-YFP in Arabidopsis. (a) Transient transformation of Arabidopsis wild-type leaves and protoplasts. Leaves from wild-type Arabidopsis plants were infiltrated with *A. tumefaciens* carrying the fl2-YFP construct. Protoplasts derived from wild-type Arabidopsis heterotrophic cell suspension cultures were transiently transformed with the fl2-YFP construct. (b) Stable transformation of Arabidopsis seedlings. Observations were carried out in roots from Arabidopsis seedlings stably transfected with the fl2-YFP construct. Fluorescence microscopy images with filters for YFP, chlorophyll/ER-Tracker Red, plus an overlay of the two channels are shown. In (a) upper row, the fl2-YFP signal shows a reticular distribution compatible with ER profiles, caging chloroplasts (highlighted in red by chlorophyll autofluorescence). In (b) lower row, the fl2-YFP signal well matches the red fluorescent signal of the ER marker ER-Tracker Red. Bars, 5 μm (a) and 10 µm (b).

**Figure S4.** Immunofluorescence analyses of Arabidopsis cell suspension cultures stably expressing fl2-AEQmut. After fixation, cells were incubated with the anti-aequorin antibody followed by secondary antibody conjugated to Alexa Fluor 594 (b). As negative control, cells were incubated with secondary antibody only (a). A red fluorescent signal compatible with the distribution of cortical ER was observed in the decorated sample, but not in the control. Bar, 10 μm.

**Figure S5.** *In vitro* reconstitution assays in Arabidopsis fl2-AEQmut transgenic lines. Luminescence emitted by 50 µg protein extracts from the fl2-AEQmut #6 and #10 lines (light blue columns) was measured. A wild-type line was used as negative control (grey column). Data are the means ± SE of ≥ 8 independent experiments. Bars labelled with different letters differ significantly (*P* < 0.05, Student’s *t* test).

**Figure S6.** *In vivo* reconstitution assays in Arabidopsis fl2-AEQmut transgenic lines. Seedlings were reconstituted in the dark with 5 μM wild-type coelenterazine (dark colour bars on the left) or coelenterazine *n* (light colour bars on the right) in 600 µM EGTA for different incubation times: overnight (16 h, purple), 8 h (red), 4 h (orange), 2 h (yellow, green, light blue and blue). Prior to reconstitution, some samples were infiltrated with the ionophore A23187 (10 μM, green), the ECA inhibitor CPA (50 μM, light blue) or both (blue). The reconstitution protocol employing 10 min pre-treatment with CPA and 2 h incubation with wild-type coelenterazine provided the highest luminescence yield. Data are the means ± SE of ≥6 different seedlings derived from three independent growth replicates. Bars labelled with different letters differ significantly (*P* < 0.05, Student’s *t* test).

**Figure S7.** Effect of the fl2-AEQmut reconstitution protocol on Arabidopsis cell viability. Arabidopsis suspension-cultured cells derived from the transgenic lines (mid-exponential phase) were extensively washed in Ca^2+^-free culture medium and then incubated with 50 µM CPA and 600 µM EGTA for 2 h and 10 min (light blue column). Control cells (white column) were incubated with cell culture medium only. The 100% value corresponds to cells treated for 15 min at 100°C (black column). Data are the means ± SE of 3 experiments. Bars labelled with different letters differ significantly (*P* < 0.05, Student’s *t* test).

**Figure S8.** Basal [Ca^2+^]_ER_ levels are independent of the concentration of CaCl_2_ used in the refilling step. [Ca^2+^]_ER_ refill trials were performed in Arabidopsis fl2-AEQmut seedlings after the *in vivo* reconstitution protocol. Different concentrations of CaCl_2_ (1 mM, light blue trace; 2 mM, blue trace; 5 mM, violet trace; 10 mM, pink trace) led to similar basal [Ca^2+^]_ER_ levels. The white arrowhead indicates the time of injection (100 s). Representative traces out of 3 independent experiments are shown.

**Figure S9.** Monitoring of [Ca^2+^]_ER_ dynamics in response to stimuli of biotic nature. Ca^2+^ assays were performed in fl2-AEQmut Arabidopsis seedlings. After the administration of 1 mM CaCl_2_ (at 100 s, white arrowhead) to restore the basal [Ca^2+^]_ER_, seedlings were challenged (light blue traces) at 300 s (arrowhead) with: (a) 1 μM flg22; (b) 1 μg ml^-1^ short chain chitin oligomers (COs); (c) 20 μg ml^-1^ oligogalacturonides (OGs). Grey traces refer to ER Ca^2+^ dynamics in response to administration (at 300 s) of H_2_O (control). Data are the means (solid lines) ± SE (shading) of ≥3 independent experiments.

**Table S1.** List of primers used to target the fl2-fused probes to the ER.

**Movie S1.** Time-lapse confocal microscopy of a cortical sector of an Arabidopsis leaf epidermal cell stably expressing fl2-YFP. fl2-YFP labels a dynamic network of tubules, showing the typical, rapid remodelling of the ER membranes.
